# Molecular Characterization of N-glycan Degradation and Transport in *Streptococcus pneumoniae* and Its Contribution to Virulence

**DOI:** 10.1371/journal.ppat.1006090

**Published:** 2017-01-05

**Authors:** Melissa Robb, Joanne K. Hobbs, Shireen A. Woodiga, Sarah Shapiro-Ward, Michael D. L. Suits, Nicholas McGregor, Harry Brumer, Hasan Yesilkaya, Samantha J. King, Alisdair B. Boraston

**Affiliations:** 1 Department of Biochemistry and Microbiology, University of Victoria, Victoria, British Columbia, Canada; 2 Center for Microbial Pathogenesis, The Research Institute at Nationwide Children’s Hospital, Columbus, Ohio, United States of America; 3 Michael Smith Laboratories and Department of Chemistry, University of British Columbia, 2185 East Mall, Vancouver, British Columbia, Canada; 4 Department of Infection, Immunity & Inflammation, University of Leicester, Leicester, United Kingdom; University of Birmingham, UNITED KINGDOM

## Abstract

The carbohydrate-rich coating of human tissues and cells provide a first point of contact for colonizing and invading bacteria. Commensurate with N-glycosylation being an abundant form of protein glycosylation that has critical functional roles in the host, some host-adapted bacteria possess the machinery to process N-linked glycans. The human pathogen *Streptococcus pneumoniae* depolymerizes complex N-glycans with enzymes that sequentially trim a complex N-glycan down to the Man_3_GlcNAc_2_ core prior to the release of the glycan from the protein by endo-β-N-acetylglucosaminidase (EndoD), which cleaves between the two GlcNAc residues. Here we examine the capacity of *S*. *pneumoniae* to process high-mannose N-glycans and transport the products. Through biochemical and structural analyses we demonstrate that *S*. *pneumoniae* also possesses an α-(1,2)-mannosidase (SpGH92). This enzyme has the ability to trim the terminal α-(1,2)-linked mannose residues of high-mannose N-glycans to generate Man_5_GlcNAc_2_. Through this activity SpGH92 is able to produce a substrate for EndoD, which is not active on high-mannose glycans with α-(1,2)-linked mannose residues. Binding studies and X-ray crystallography show that NgtS, the solute binding protein of an ABC transporter (ABC_NG_), is able to bind Man_5_GlcNAc, a product of EndoD activity, with high affinity. Finally, we evaluated the contribution of EndoD and ABC_NG_ to growth of *S*. *pneumoniae* on a model N-glycosylated glycoprotein, and the contribution of these enzymes and SpGH92 to virulence in a mouse model. We found that both EndoD and ABC_NG_ contribute to growth of *S*. *pneumoniae*, but that only SpGH92 and EndoD contribute to virulence. Therefore, N-glycan processing, but not transport of the released glycan, is required for full virulence in *S*. *pneumoniae*. To conclude, we synthesize our findings into a model of N-glycan processing by *S*. *pneumoniae* in which both complex and high-mannose N-glycans are targeted, and in which the two arms of this degradation pathway converge at ABC_NG_.

## Author Summary

*Streptococcus pneumoniae* (pneumococcus) is a bacterium that causes extensive morbidity and mortality in humans. Vaccines and antibiotics are effective forms of prevention and treatment, respectively, but present challenges as it is a constant race to vaccinate against the enormous and ever evolving pool of different serotypes of the bacterium while resistance to antibiotics continues to trend upwards. It is thus necessary to better understand the molecular aspects of the host-pneumococcus interaction in order to inform the potential generation of alternative treatment strategies. *S*. *pneumoniae* relies on its ability to process the carbohydrates presented on the surface of host cells for full-virulence. In this study, we examine the capability of the bacterium to process high-mannose N-linked sugars, a heretofore unknown ability for *S*. *pneumoniae*. The results show that the pneumococcal genome encodes enzymes capable of processing these sugars and that, remarkably, the initiating reaction performed by an enzyme that removes terminal α-(1,2)-linked mannose residues is critical to virulence in a mouse model. This study illuminates an extensive pathway in *S*. *pneumoniae* that targets N-linked sugars and is key to the host-pathogen interaction, therefore revealing a potential target for therapeutic intervention.

## Introduction

The covalent attachment of carbohydrates to macromolecules, such as proteins and lipids, to form glycoconjugates is one of the most abundant modifications of molecules in nature. In the human body, the glycans displayed on glycoproteins and glycolipids in and on the outer surface of cells help to form the complex carbohydrate-rich glycocalyx that surround cells and contribute to key functions such as cell-cell interactions, cell signaling, and physical protection [1]. Given the vital purposes of glycans, and specifically glycoproteins, it is perhaps not surprising that commensal microbes and microbial pathogens have developed mechanisms to take advantage of the carbohydrate-rich environment in the human body to pave the way for both colonization and, in the case of pathogens, infection [2].

The attachment of glycans to the side chains of asparagine residues, so-called N-glycosylation, is the most common form of protein glycosylation and, therefore, one of the most frequent forms of post-translational modification to proteins [3]. N-glycans are essential to the proper function of glycoproteins by providing a range of biochemical properties important to the folding, stability and activity of their protein scaffold, thus enabling these glycoconjugates to assume their essential roles in a wide range of physiological processes in the human body [4]. Indeed, glycoproteins in the human body with this modification contribute to an abundance of critical biological roles including structural functions, protection of tissues, immune response, hormone signaling, cell and molecule attachment, blood coagulation, and many more.

N-glycans are built on a common α-D-Man-(1→6)-[α-D-Man-(1→3)]-β-D-Man-(1→4)-β-D-GlcNAc-(1→4)-β-D-GlcNAc core (Man_3_GlcNAc_2_), with the terminal N-acetylglucosamine (GlcNAc) residue linked to the side chain of asparagine through an amide bond (S1 Fig). The terminal mannose residues can be modified with additional monosaccharides, often GlcNAc, sialic acid, and galactose, to form complex N-glycans (S1 Fig); with additional mannose residues to form high-mannose glycans (S1 Fig); or a combination of the two to form hybrid glycans. Several bacteria able to colonize and/or cause infections in humans are noted to have the ability to depolymerize N-glycans. For example, *Bacteroides fragilis*, *Capnocytophaga canimorsus*, *Enterococcus faecalis*, *Streptococcus pneumoniae*, *Streptococcus oralis*, and *Streptococcus pyogenes* have all been postulated to rely to some extent on their ability to degrade N-glycans to assist in the infection process [5–11].

In Gram-negative bacteria, the importance of N-glycan degrading systems to virulence is best supported by studies of *C*. *canimorsus* that identified N-glycan degrading glycoside hydrolases and revealed that complete deletion of a locus encoding the ability to degrade N-glycans decreased fitness of the bacterium in a mouse model [10,11]. The proposed model of complex N-glycan metabolism in *C*. *canimorsus* involves release of the glycan from a glycoprotein by an endo-β-N-acetylglucosaminidase, transport of the glycan to the periplasm by TonB-dependent transport, and depolymerization of the imported glycan in the periplasm by a sialidase, β-galactosidase, β-N-acetylhexosaminidase, and α-mannosidase [11]. A similar model, which is presently the most thoroughly validated in any system, is proposed for the degradation of high-mannose glycans by another Bacteroidete: the Gram-negative commensal human gut microbiome bacterium *Bacteroides thetaiotaomicron* [12]. In this pathway, however, only periplasmic α-mannosidases are required to complete depolymerization of high-mannose glycans [12]. Unlike *C*. *canimorsus*, where N-glycan degradation is linked to pathogenesis, *B*. *thetaiotaomicron* appears to utilize its high-mannose degrading pathway when polysaccharides are limited in the diet of the host and the bacterium must switch to host glycans as a nutrient source [12,13].

Despite the identification of enzymes key to the process of N-glycan degradation pathways in Gram-positive bacteria long before the study of analogous pathways in Gram-negative bacteria, the molecular and functional characterization of these pathways in Gram-positive bacteria is lagging behind. At present, the best model for N-glycan degradation by a Gram-positive bacterium is *S*. *pneumoniae*, which is a globally important pathogen responsible for high morbidity and mortality resulting from pneumonia and meningitis [14]. Enzymes from *S*. *pneumoniae* with glycoside hydrolase activities on the linkages in complex N-glycans (i.e. sialidase, β-galactosidase, β-N-acetylglucosaminidase, and endo-β-N-acetylglucosaminidase) were identified over 40 years ago [15–17]. More recently, the *exo*-α-sialidase (NanA), the *exo*-β-galactosidase (BgaA), and the *exo*-β-*N*-acetylglucosaminidase (StrH), all of which are attached to the outer cell-surface of the bacterium, were shown to have a necessarily sequential activity on the arms of complex N-glycans [18] and, through this activity, contribute to nutrient acquisition and suppression of the host innate immune response [9,19]. The *endo*-β-*N*-acetylglucosaminidase EndoD, which is also a cell-surface protein, cleaves the chitobiose core of mannose-containing N-linked glycans with five or fewer mannose residues to release the glycan from proteins [20]. Consistent with the theoretically critical position of this enzyme in an N-glycan metabolising pathway, the importance of EndoD to the virulence of *S*. *pneumoniae* is suggested by signature-tagged mutagenesis (STM) experiments, but its full importance to the host-bacterium interaction remains unclear. More recently, an α-mannosidase from *S*. *pneumoniae* was structurally and functionally demonstrated to be an N-glycan active α-(1,6)-mannosidase (SpGH125), providing the first evidence that the genome of this bacterium encodes the biochemical capability of depolymerising the mannose portion of the N-glycans [21]. The gene encoding this enzyme was also indicated by STM experiments to have potential involvement in virulence [22]. Thus, a picture is beginning to emerge of a pathway in this Gram-positive bacterium that furnishes the capacity for concerted degradation of N-glycans and that is important to the interaction of *S*. *pneumoniae* with its host.

A full understanding of N-glycan degradation by *S*. *pneumoniae*, however, is lacking as several key issues remain unresolved that prevent the assembly of a complete and reliable model for this pathway and its role in *S*. *pneumoniae* virulence. Towards improving our understanding of the N-glycan degradation pathway, we have used bioinformatics to identify a carbohydrate-processing locus (CPL) and other co-occurring genes that together comprise ORFs encoding proteins putatively involved in N-glycan processing. Known components of this system include EndoD and SpGH125 [21,23,24]. Here we characterize SpGH92 (TIGR4 locus tag SP_2145) as an α-mannosidase that trims the terminal α-(1,2)-linked mannose residues on high-mannose glycans to generate a substrate for EndoD. We also identify a solute binding protein (SBP; NgtS) from an ABC transporter (ABC_NG_) that binds soluble N-glycans and, through X-ray crystallography, reveal the molecular basis of its interaction with these branched glycans. The binding activity of NgtS is consistent with binding the catalytic product(s) of EndoD activity. *S*. *pneumoniae* strains with deletions in the genes encoding for SpGH92 and EndoD, but not ABC_NG_, were attenuated in a mouse model of virulence revealing that N-glycan processing, but not transport, is necessary for full virulence of *S*. *pneumoniae* TIGR4 in an animal model. These new results, which we synthesize into a complete model of N-glycan processing by *S*. *pneumoniae*, provides a valuable molecular framework for understanding N-glycan processing in *S*. *pneumoniae*, related streptococci, and likely other Gram-positive bacteria. Furthermore, they also highlight the potential for targeting N-glycan degradation as a therapeutic tactic for combating *S*. *pneumoniae*.

## Results

### Identification of a carbohydrate-processing locus (CPL)

**Fig 1:**
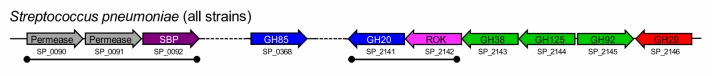
Organization and conservation of a Carbohydrate Processing Locus (CPL) and other accessory proteins predicted to be associated with N-glycan processing. Genetic organization of a CPL and other accessory ORFs present in strains of *S*. *pneumoniae* whose protein products are putatively associated with N-glycan processing. The CPL consists of a characterized α-(1,6)-mannosidase (SpGH125) [21], two putative α-mannosidases (SpGH38 and SpGH92), a predicted α-fucosidase (GH29), a putative sugar kinase (ROK) and a known *exo*-β-hexosaminidase (SpGH20C) [28]. Other conserved proteins predicted to be associated with N-glycan processing include a characterized endo-β-N-acetylglucosaminidase (SpGH85 or EndoD) that is active on the core of N-linked glycans [24] and an ABC transporter (ABC_NG_) that consists of a predicted solute binding protein (NgtS) and two putative permeases (NgtP1 and NgtP2). Locus tags shown underneath ORFs relate to the TIGR4 genome; black bars underneath ORFS indicate genes that are predicted to be co-transcribed. For the majority of the ORFs, coloring corresponds to the type of configured sugar acted on by the hydrolase: fucose (red), glucose (blue) and mannose (green).

The region of the *S*. *pneumoniae* TIGR4 genome comprising ORFs SP_2141 to SP_2146 is part of the described “core” *S*. *pneumoniae* genome [25]. Moreover, all of the genes in this locus except SP_2141 have shown up in multiple STM and microarray studies as putative virulence factors [22,25–27], leading it to be considered an important component of the *S*. *pneumoniae*-host interaction. SP_2141 and SP_2144 have been functionally studied and shown to be glycoside hydrolases (GH) with *exo*-β-*N*-acetylhexosaminidase and α-(1,6)-mannosidase activity, respectively [21, 28]. GHs are classified into >133 amino acid sequence-based families and SP_2144 falls into GH family 125, thus it is referred to as SpGH125, whereas SP_2141 (SpGH20C) falls into GH family 20. Consistent with the presence of this demonstrated carbohydrate-active enzyme in this genomic locus, an annotation of the additional ORFs in this region based on the GH classification shows them to encode proteins falling into GH families 29 (SP_2146), 38 (SP_2143), and 92 (SP_2145); the fifth ORF encodes a ROK (repressor, ORF, sugar kinase) family protein. Based on these GH family annotations, we can make hypothetical carbohydrate-active enzyme annotations of α-fucosidase (SP_2146/SpGH29), and α-mannosidase activities (SP_2143/SpGH38 and SP_2145/SpGH92) (Table 1). The concentration of genes encoding for known and putative carbohydrate-processing enzymes has led us to refer to this six-gene cluster as a CPL (Carbohydrate-Processing Locus; Fig 1).

**Table 1:** *S*. *pneumoniae* proteins encoded by the CPL or co-occurring with the CPL in *S*. *pneumoniae* and other streptococci.

Locus tag	Protein name	Known or putative activity	Virulence association
^a^SP_2141	GH20C	*Exo*-β-hexosaminidase [28]	-
^a^SP_2142	ROK	Putative sugar kinase	STM [22,26]
^a^SP_2143	GH38	Putative *exo*-α-(1,3)-mannosidase	STM [26]
^a^SP_2144	GH125	*Exo*-α-(1,6)-mannosidase [21]	STM [22]
^a^SP_2145	GH92	Putative *exo*-α-(1–2)-mannosidase	STM [22,26,29]
^a^SP_2146	GH29	Putative *exo*-α-fucosidase	STM [22,26]
SP_0498	EndoD (GH85)	N-glycan *endo*-β-N-acetylglucosaminidase [20,24]	STM [26,29]
SP_0090	NgtP1	Putative ABC transporter transmembrane permease	-
SP_0091	NgtP2	Putative ABC transporter transmembrane permease	-
SP_0092	NgtS	Putative ABC transporter solute binding protein	STM [26]

^a^These genes comprise the CPL.

Abbreviation: Signature-tagged mutagenesis (STM).

Family 125 glycoside hydrolases are quite widely distributed in bacteria and fungi [30]. Furthermore, when a gene encoding a GH125 enzyme is present in an organism it very often occurs as a pair with a gene encoding a GH38 enzyme [21]. These pairs have been hypothesized to encode complementary α-(1,6)-mannosidase (GH125) and α-(1,3)-mannosidase (GH38) activities that enable depolymerization of the N-glycan cores in the Man_3_ to Man_5_ motifs [21]. Using this gene pair as a marker for the CPL, we searched for the most similar homologs in other bacteria. This resulted in the identification of very similar loci in a number of streptococci and other Firmicutes (S2 Fig). This *ad hoc* analysis suggested that GH92, GH20, ROK, and GH29 co-occur with the GH125 and GH38 pair. The co-occurrence and functional association of these proteins was more quantitatively supported by an analysis with STRING_10_ [31], which for the pairwise analysis all the combinations of these six enzymes yielded scores of >0.8 for potential functional association. Notably, this analysis also identified an ABC transport system, encoded by locus tags SP_0090 to SP_0092 (scores >0.7), and EndoD (scores >0.8) as possibly being functionally associated with the CPL. Indeed, in 8 of the 10 loci we identified a homologous ABC transporter that is also genomically associated with the CPL, further implying linked functional roles. Though an EndoD homolog is only sometimes incorporated into the locus, it appears to frequently co-occur with the locus in Firmicutes. Thus, this bioinformatics analysis pointed to a putative functional linkage between the CPL, EndoD, and an ABC transporter encoded by locus tags SP_0090 to SP_0092, which we will refer to as ABC_NG_. On the basis of this bioinformatics analysis we hypothesized that SpGH92, EndoD, and ABC_NG_ are functionally associated, serving to assist in processing and transporting N-glycans as part of the host-pathogen interaction.

### SpGH92 (SP_2145) from the CPL is an α-(1,2)-mannosidase that trims high-mannose N-glycans, generating a substrate for EndoD

**Fig 2:**
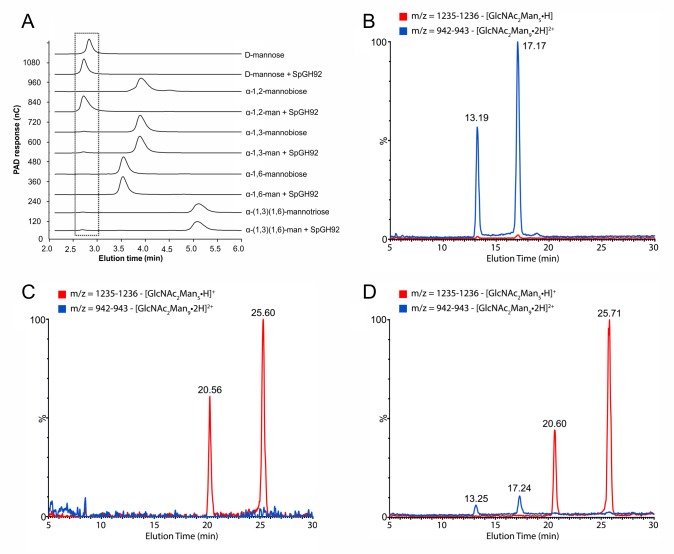
α-(1,2)-mannosidase activity of SpGH92. (A) Activity of SpGH92 against α-(1,2), α-(1,3) and α-(1,6)-mannobiose and α-(1,3)(1,6)-mannotriose observed by HPAEC-PAD. Dashed box indicates elution of mannose. (B-D) Activity of SpGH92 on a high-mannose N-glycan (Man_9_GlcNAc_2_) observed by LC-MS. (B) Extracted ion chromatogram (EIC) of Man_9_GlcNAc_2_ standard, indicating resolution of both anomers with an *m/z* value of 942.3304 [M+2H]^2+^ (expected 942.3290, Δ*m/z* = 1.5 ppm). (C) EIC of Man_5_GlcNAc_2_ standard, indicating resolution of both anomers with an *m/z* value of 1235.4322 [M+H]^+^ (expected 1235.4400, Δ*m/z* = 6.3 ppm). (D) EIC of Man_9_GlcNAc_2_ treated with SpGH92 showing that the enzyme trims the α-(1,2)-linked mannose residues of Man_9_GlcNAc_2_, resulting in the formation of Man_5_GlcNAc_2_.

The protein product of SP_2145 falls into GH family 92; characterized members of this family exhibit α-mannosidase activity with specificity for either α-(1,2), α-(1,3) or α-(1,6) linkages. Given the occurrence of SpGH92 within the CPL, the known α-(1,6)-mannosidase activity of SpGH125 [21] and the likely α-(1,3)-mannosidase activity of SpGH38 (given that its close homolog, SpyGH38 from *S*. *pyogenes*, exhibits this activity [32]), we hypothesized that SpGH92 is an α-(1,2)-mannosidase. To test this, the activity of recombinant SpGH92 on various mannose-containing oligosaccharide motifs found in N-linked glycans was examined by HPAEC-PAD analysis (Fig 2A). Of α-(1,2)-mannobiose, α-(1,3)-mannobiose, α-(1,6)-mannobiose and α-(1,3)(1,6)-mannotriose, SpGH92 was only able to cleave α-(1,2)-mannobiose into its constituent mannose residues. Furthermore, SpGH92 activity was only observed when Ca^2+^ was provided in the reaction mixture, consistent with the Ca^2+^ dependence displayed by other family members [33]. Using an LC-MS approach, we also observed that treatment of Man_9_GlcNAc_2_ with SpGH92 produced a glycan having a mass consistent with Man_5_GlcNAc_2_ (Fig 2B–2D). Together these results reveal that SpGH92 is an α-(1,2)-mannosidase that is active on the α-(1,2)-mannose decorations of high-mannose N-glycans.

**Fig 3:**
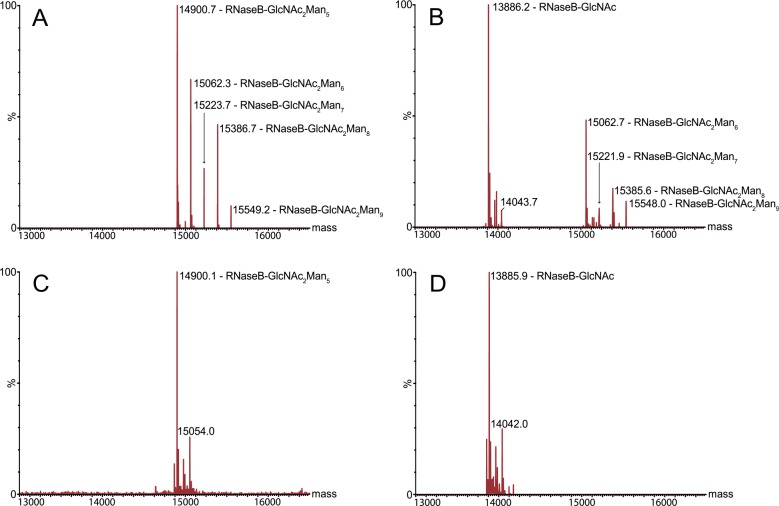
Deglycosylation of RNase B by SpGH92 and EndoD. (A) Reconstructed mass spectrum of native RNaseB. The following glycoforms are observed: RNaseB-GlcNAc_2_Man_5_ (exp: 14899.4 Da, obs: 14900.7 Da), RNaseB-GlcNAc_2_Man_6_ (exp: 15061.6 Da, obs: 15062.3 Da), RNaseB-GlcNAc_2_Man_7_ (exp: 15223. Da, obs: 15223.7 Da), RNaseB-GlcNAc_2_Man_8_ (exp: 15386.0 Da, obs: 15386.7 Da) and RNaseB-GlcNAc_2_Man_9_ (exp: 15548.2 Da, obs: 15549.2 Da). (B) EndoD cleaves the chitobiose core of Man_5_GlcNAc_2_, yielding RNaseB-GlcNAc (exp: 13885.2 Da, obs: 13886.2 Da), but cannot act on the Man_6_-Man_9_ glycoforms. (C) SpGH92 trims the Man_6_-Man_9_ glycoforms down to the RNaseB-GlcNAc_2_Man_5_ glycoform (exp: 14899.4 Da, obs: 14900.1 Da). (D) Together, SpGH92 and EndoD act on all glycoforms of RNase B to produce exclusively RNaseB-GlcNAc (exp: 13885.2 Da, obs: 13885.9 Da).

In the processing of N-glycan by *S*. *pneumoniae*, EndoD acts to cleave the chitobiose core, releasing the glycan from the protein; however, it is only able to carry out this function on N-glycans that carry five or fewer mannose residues (i.e. Man_5_GlcNAc_2_ or smaller) [20]. Therefore, we speculated that the role of SpGH92 may be to trim the terminal α-(1,2)-linked mannose residues from high-mannose N-glycans in order to make it accessible to EndoD. RNase B is a model glycoprotein that has a single, high-mannose N-linked glycosylation site [34]. As such, we used it as a model substrate to test the sequential ability of SpGH92 and EndoD to degrade high-mannose N-glycan. Initially, we used SDS-PAGE to observe the solo and combined activities of SpGH92 and EndoD on RNase B, which showed that both SpGH92 and EndoD were required to completely convert RNase B into a low molecular weight form (S3 Fig). We then used mass spectrometry on intact RNaseB, which was able to resolve the Man_5_GlcNAc_2_ up to Man_9_GlcNAc_2_ glycoforms of this protein [34] (Fig 3A), to investigate the activity of these two enzymes. Treatment of RNase B with only EndoD confirmed that this enzyme is only able to act on the Man_5_GlcNAc_2_ glycoform, producing a protein species with a single GlcNAc on it, while leaving the larger glycoforms intact (Fig 3B). Treatment of RNase B with SpGH92 eliminated the Man_6_GlcNAc_2_-Man_9_GlcNAc_2_ glycoforms producing a single Man_5_GlcNAc_2_ species (Fig 3C). Together, SpGH92 and EndoD fully deglycosylated RNase B (Fig 3D). These findings illustrate the sequential nature of high-mannose N-glycan deglycosylation performed by SpGH92 and EndoD.

### X-ray crystal structure of SpGH92

**Fig 4:**
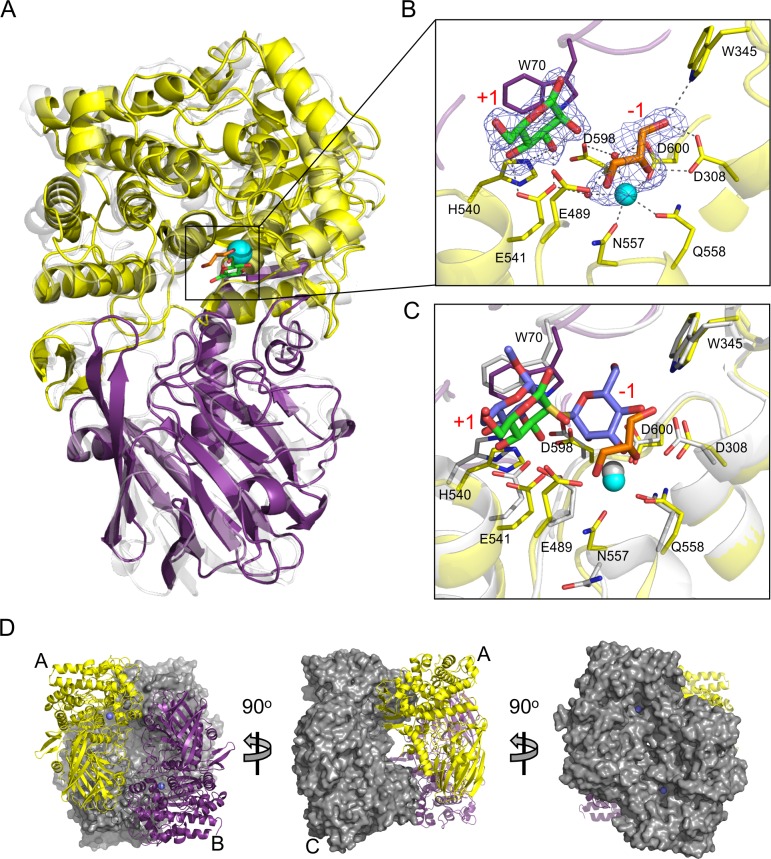
X-ray crystal structure of SpGH92. (A) The overall structure of SpGH92 with the (α/α)_6_ barrel domain colored in yellow and the β-sandwich domain colored in purple. The bound calcium is shown as a cyan sphere, the bound glycerol as orange sticks and the bound mannose as green sticks. The structure is overlaid with that of Bt3990, which is shown in transparent grey. (B) The active site of SpGH92. The calcium, glycerol, and mannose are shown as in panel A. The blue mesh is the σ_a_-weighted F_o_-F_c_ electron density map (contoured at 3σ) for the glycerol and mannose. Relevant side chains involved in ligand binding and catalysis are shown as sticks. (C) The active site of SpGH92 overlaid with the active site of Bt3990 (PDB ID 2WW3). SpGH92 is as shown in panel B. Bt3990 is shown in grey with active site residues shown as sticks. The methyl-2-S-(α-D-mannopyranosyl)-2-thio-α-D-mannopyranoside bound in the Bt3990 active site is shown as blue sticks. In panels B and C, the subsites in the active site are labeled in red text. (D) The arrangement of the SpGH92 tetramer shown from three perspectives. One pair of monomers is shown in cartoon representation and the other pair as surfaces. The bound calcium atoms are shown as blue spheres to mark the location of the active site. Monomers A, B, and C are labeled to indicate the A/B and A/C dimers making up the D2 symmetry of the tetramer.

To further probe the finding that SpGH92 is an α-(1,2)-mannosidase, we pursued structural studies by X-ray crystallography. Crystals of the protein soaked in a solution containing α-(1,2)-mannobiose gave a diffraction data set to 2.15 Å resolution with a space group of P2_1_2_1_2_1_ (Table 2; PDB ID 5SWI). The structure was determined by molecular replacement using *B*. *thetaiotaomicron* GH92, Bt3990 (PDB ID 2WVX) as a search model. The final refined model contained four SpGH92 molecules in the asymmetric unit with each monomer comprising two domains: an N-terminal β-sandwich domain and a larger C-terminal (α/α)_6_ barrel domain (Fig 4A), which is a conserved and widespread fold adopted by other GHs from families 8, 15, 37, 48, 63, 65, and 125 [30]. The N-terminal domain is joined to the C-terminal (α/α)_6_ barrel by a helix-strand-helix motif. The strand in this linker region pairs with four additional strands at the C-terminus of the protein to create a 5-stranded antiparallel β-sheet that packs against one edge of the (α/α)_6_ barrel. Each monomer binds a single metal atom, which on the basis of B-factor analysis after refinement, and coordination geometry, was modeled as a Ca^2+^ atom. This atom is bound in a relatively deep cavity present at the centre of the (α/α)_6_ barrel (Fig 4B) where electron density consistent with a single mannose residue, resulting from the hydrolysis of the α-(1,2)-mannobiose the crystal was soaked in, and a glycerol molecule was also observed (Fig 4B). The glycerol molecule was coordinated by the Ca^2+^ atom as well as additional direct and water mediated hydrogen bonds. The mannose residue packs against the face of the Trp70 sidechain in a classic pyranose ring-aromatic amino acid side chain interaction; additional direct hydrogen bonds are made between amino acid side chains in the active site and the O4 and O3 hydroxyls of the mannose (Fig 4B).

**Table 2:** Data collection and refinement statistics for SpGH92 and NgtS.

	SpGH92 Mannose	NgtS NaI	NgtS Man_1_GlcNAc	NgtS Man_5_GlcNAc
**Data collection statistics**				
Beamline	SSRL BL7-1	Home source	SSRL BL9-2	SSRL BL9-2
Wavelength	0.9753	1.54187	1.04007	0.98005
Spacegroup	P2_1_2_1_2_1_	P2_1_	P1	P2_1_
Cell dimensions: *a*, *b*, *c* (Å)	130.0, 161.7, 208.4	60.91, 40.20, 93.89	39.99, 50.47, 107.89	104.18, 11.60, 105.84
Resolution (Å)	40.00–2.15 (2.19–2.15)*	21.80–2.10 (2.21–2.10)*	45.95–3.00 (3.16–3.00)*	48.37–1.73 (1.82–1.73)*
R_merge_	0.103 (0.819)*	0.126 (0.503)*	0.137 (0.459)*	0.036 (0.397)*
I/σI	14.4 (2.6)*	19.5 (5.1)*	6.6 (2.3)*	25.4 (4.0)*
Completeness (%)	99.9 (98.7)*	99.8 (99.6)*	97.0 (97.0)*	99.9 (99.9)*
Redundancy	7.5 (7.4)*	10.9 (10.5)*	2.2 (2.1)*	4.7 (4.6)*
**Refinement statistics**				
Total reflections	1786138	291180 (40192)*	35594 (5086)*	1095333 (158575)*
Unique reflections	237386	26624 (3833)*	16399 (2413)*	234916 (34209)*
R_work_/R_free_	19.7/22.4	21.1/26.1	22.9/28.1	18.2/22.0
No. of atoms				
Protein	21991 (4 molecules)	3491 (1 molecule)	6884 (2 molecules)	14283 (4 molecules)
Ligands	4 (CA), 48 (BMA), 96 (GOL)	21 (IOD)	52 (CHO), 2 (BR)	280 (CHO), 35 (CD)
Solvent	1364 (H_2_O)	227 (H_2_O)	51 (H_2_O)	2149 (H_2_O)
Average B-factors (Å^2^)				
Protein	36.3	20.1	39.5	29.0
Ligands	23.3 (CA), 39.0 (BMA), 49.5 (GOL)	38.3 (IOD)	40.2 (CHO), 65.4 (BR)	20.4 (CHO)
Solvent	36.6 (H_2_O)	19.2 (H_2_O)	18.9 (H_2_O)	20.4 (H_2_O), 40.3 (CD)
R.m.s. deviations				
Bond lengths (Å)	0.009	0.010	0.004	0.019
Bond angles (°)	1.232	1.418	0.835	1.893
Ramachandran				
Preferred (%)	97.4	96.7	93.5	97.8
Allowed (%)	2.2	3.1	5.8	2.0
Disallowed (%)	0.3 (8 residues)	0.2 (1 residue)	0.7 (5 residues)	0.2 (4 residues)

*Parentheses indicate values for the high-resolution reflection bins.

SpGH92 displays the same general fold as the available structures of three other GH92 structures from *B*. *thetaiotaomicron* (Bt3990, PDB ID 2WVX; and Bt2199, PDB ID 2WVY) and *Cellulosimicrobium cellulans* (CcGH92_5, PDB ID 2XSG) with overall root mean square deviations (rmsd) of less than 2.3 Å. The most similar enzyme to SpGH92 is Bt3990 [33], a periplasmic mannosyl-oligosaccharide α-(1,2)-mannosidase, which has an amino acid sequence identity of 30% and an rmsd of 1.8 Å over 689 (of 693) matched Cα atoms with SpGH92. An overlay of SpGH92 with the structure of Bt3990 solved in complex with a non-hydrolyzable thio-linked α-(1,2)-mannobiose shows the active site of Bt3990 to be highly conserved with SpGH92 (Fig 4C). Furthermore, the mannose present in the SpGH92 complex overlaps with the mannose residue in the +1 subsite of Bt3990 while the glycerol bound in the SpGH92 active site approximates the positions of carbons 2, 3, and 4 in the mannose residue present in the -1 subsite of Bt3990. GH92 enzymes use an inverting catalytic mechanism, whereby the glycosidic bond is hydrolyzed with inversion of the stereochemistry at the anomeric carbon by a single displacement mechanism; this mechanism employs two amino acid side chains that act as a general acid and a Brønstead base, the latter of which activates a water molecule to nucleophilically attack the anomeric carbon [33]. In Bt3990 the acid residue is Glu533 and the base is Asp644 [33], which are homologous to Glu489 and Asp600, respectively, in SpGH92. The coordination of the calcium atom, which in Bt3990 is involved in recognizing O2 and O3 of the mannose residues in the -1 subsite, is also conserved between the two enzymes, completing the catalytic machinery of the proteins (Fig 4C). Additional residues providing polar and non-polar interactions in the -1 subsite are also retained between the two enzymes. The primary difference between the two enzymes in this -1 subsite is in the SpGH92 loop comprising 352-Gly-Met-Met-Pro-Gly-356, which in Bt3990 is 392-Gly-Cys-Met-Val-Gly-396. In Bt3990 this loop is positioned above the active site with the methionine side chain placed over the mannose reside in the -1 subsite. In SpGH92 this loop is slightly retracted from the active site in three of the monomers in the asymmetric unit, while in the fourth monomer the loop could be modeled in the retracted conformation as well as an engaged conformation. Thus, it appears that this loop in SpGH92 is somewhat mobile and it is possible that substrate binding could trigger movement and ordering of this loop to more fully engage the substrate when a sugar residue occupies the -1 subsite, rather than a glycerol molecule, as it is in our product complex. Bt3990 also possesses a +1 subsite that is responsible for binding the mannose residue of the leaving group. This subsite, which comprises Trp88, Glu585, and His584, provides the α-(1–2) mannose specificity in this enzyme; the architecture of this subsite is completely conserved in SpGH92 with the trio of analogous residues being Trp70, Glu541, and His540. Thus, the conserved architecture of the substrate binding sites in SpGH92 with that of an obligate α-(1,2)-mannosidase is consistent with both the observed activity profile of SpGH92 and the structure of it in complex with mannose, thus further supporting the assignment of this enzyme as a specific α-(1,2)-mannosidase, which is an enzymatic activity previously unknown to be possessed by *S*. *pneumoniae*.

SpGH92 crystallized as a tetramer with D2 symmetry present in the asymmetric unit (Fig 4D). An analysis of this assembly with PISA suggests that formation of the tetramer results in ~9800 Å^2^ of buried surface area and is stable in solution with a calculated ΔG_dissociation_ of 22.2 kcal/mol. The two different possible dimers (e.g. an A monomer/B monomer (AB) type or an A monomer/C monomer (AC) type) making up the tetramer each resulted in calculated buried surface areas of ~2700 Å^2^ and ΔG_dissociation_ values of ~6.7 kcal/mol, thus suggesting that either type of dimer may also be stable in solution. To examine the oligomeric state of SpGH92 in solution, we used both size exclusion chromatography and dynamic light scattering. The theoretical molecular weight of the SpGH92 tetramer is 325.96 kDa. The molecular weight determined by size exclusion chromatography was 333.65 kDa (S4 Fig) and the molecular weight determined by dynamic light scattering was 325.38 (± 41.03) kDa [measured radius of 7.04 (± 0.37) nm and a polydispersity of 17.54 (± 3.96) %, n = 8, ±S.D.]. Thus, these results are consistent with SpGH92 forming a tetramer in solution as well as in the crystalline state. The arrangement of the AB type dimer is very similar to the crystallographic dimer formed by Bt3990 in multiple different crystal forms [33] indicating a general propensity for these similar GH92 enzymes to oligomerize with common features in their quaternary structures. The architecture of the SpGH92 tetramer results in a twisted cube shape with the two active sites contributed by each AB-type dimer on the opposing faces of the cube. Thus, substrate has ready access to the SpGH92 active site regardless of the potential multimerization of the protein.

### Binding properties of an N-glycan specific solute-binding protein, NgtS

The co-occurrence of the putative ABC_NG_ transporter with EndoD and N-glycan specific components of the CPL (SpGH125 and SpGH92) suggested to us that the specificity of the transporter may be complementary to these enzymes. We tackled this hypothesis through a functional and structural analysis of SP_0092, the putative cell-surface attached SBP of the ABC transporter, which we refer to as NgtS (N-glycan transport SBP). The binding properties of soluble recombinant NgtS that lacks the secretion signal peptide and lipid-anchoring motif was probed by isothermal titration calorimetry (ITC) and UV difference titrations. ITC using Man_5_GlcNAc as a ligand gave an association constant (K_a_) of 1.04 (± 0.01)×10^6^ M^−1^, which is consistent with the range of affinities previously determined for other SBPs [35–38], and a binding stoichiometry of 1.05 (±0.01), equating to the expected 1:1 protein to ligand ratio. The change in enthalpy (ΔH) and entropy (given as TΔS at 298.15 K) were determined to be -19.78 (±0.01) kcal mol^-1^ and -11.57 (±0.10) kcal mol^-1^, respectively, revealing the typical thermodynamic signature for a protein-carbohydrate interaction [38]. NgtS also bound to Man_1_GlcNAc; however, the low affinity and limited quantities of the sugar precluded binding analysis by ITC. Instead, we employed a UV difference binding analysis by taking advantage of a perturbation in the UV absorbance of the protein upon sugar binding. Used in a quantitative fashion we obtained an affinity constant of 1.2 (± 0.1) ×10^4^ M^−1^ for Man_1_GlcNAc, and thus approximately two orders of magnitude lower than what was obtained for Man_5_GlcNAc.

### X-ray crystal structure of NgtS

**Fig 5:**
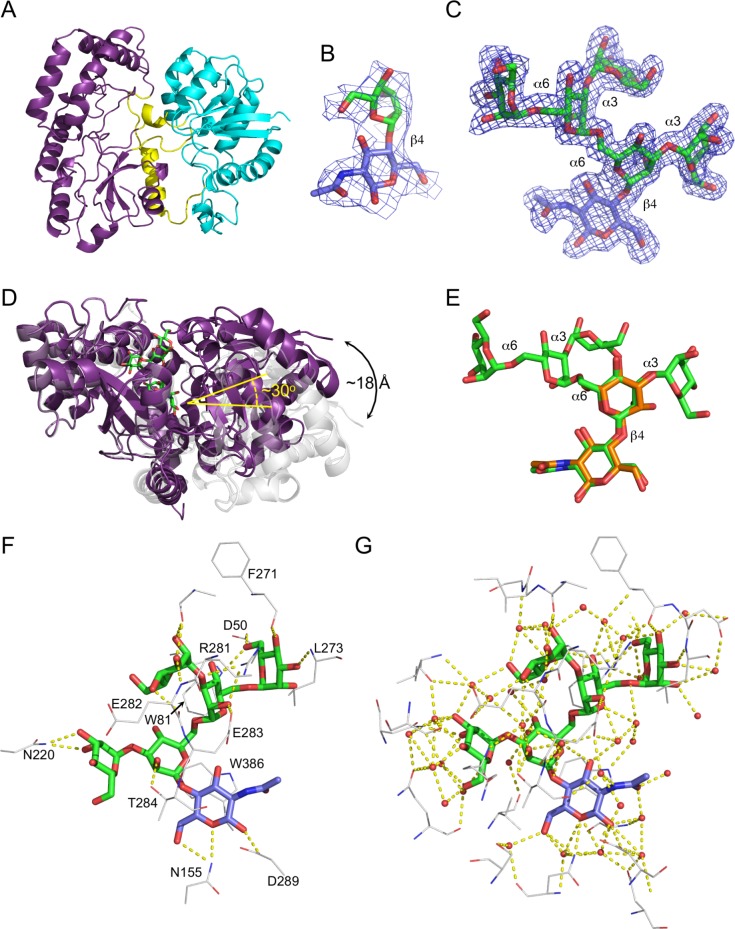
X-ray crystal structure of NgtS. (A) The overall fold of NgtS with its two domains and hinge region colored purple, cyan, and yellow, respectively. (B) The σ_a_-weighted F_o_-F_c_ electron density map (contoured at 3σ) for the ligand bound in Man_1_GlcNAc complex. (C) The σ_a_-weighted F_o_-F_c_ electron density map (contoured at 3σ) for the ligand bound in Man_5_GlcNAc complex. (D) The structure of the NgtS-Man_5_GlcNAc complex, shown in purple with green sticks for the ligand, overlaid with the apo and open form of NgtS, shown in grey. (E) An overlap of the Man_1_GlcNAc (orange sticks) and Man_5_GlcNAc (green sticks) showing the similar conformation and position of the overlapping portions. (F) Direct interactions between the NgtS binding site (residues shown as grey sticks) and the bound Man_5_GlcNAc (green sticks for mannose and blue for GlcNAc). Hydrogen bonds are shown as yellow dashes. (G) The extensive solvent network in the NgtS binding site involved in coordinating the Man_5_GlcNAc ligand. Water molecules are shown as red spheres.

Given the unprecedented ability of NgtS to bind large N-glycan fragments, we pursued analysis of its structure to understand the molecular basis of N-glycan recognition. NgtS in its unbound form formed crystals in the space group P2_1_ that were sufficiently robust to withstand soaking in high concentrations of sodium iodide to generate a halide derivative. The structure was subsequently solved by single wavelength anomalous dispersion to a resolution of 2.1 Å (Table 2; PDB ID 5SUO). The structure of NgtS is similar to that of other SBPs [39] and comprises two α/β domains separated by a hinge region (Fig 5A). Each α/β domain consists of a central β-sheet of three β-strands flanked by α-helices, with the N-terminal domain possessing an additional solvent-exposed two-stranded β-sheet.

NgtS was also co-crystallized with the ligands Man_1_GlcNAc and Man_5_GlcNAc, and diffraction data collected to 3.0 Å and 1.6 Å, respectively (PDB ID 5SWA and 5SWB). In both cases, we found clear electron density for the bound sugar, which facilitated easy modeling of the carbohydrates (Fig 5B and 5C). Despite these two complexes being obtained with different ligands and in different spacegroups, the overall conformations of NgtS were very similar (rmsd of 1.0 Å over 427 matched Cα positions); both complexes were in a “closed” conformation relative to the “open” conformation obtained in the absence of ligand. As with other SBPs [39], the ligand-binding site was found at the interface of the two domains comprising NgtS and ligand recognition involves a transition from the open, unbound form to a ligand-stabilized closed form (Fig 5D).

An overlay of the structures in complex with the two ligands showed that, within positional error of the structures, the location of Man_1_GlcNAc is the same as the corresponding Man_1_GlcNAc portion of Man_5_GlcNAc (Fig 5E). The reducing end of the carbohydrate is oriented into the base of the protein binding cleft and fully sequestered from bulk solvent with no room for additional residues present at the reducing end, consistent with our proposal that NgtS specifically binds the products of EndoD hydrolysis (i.e. having only the single non-reducing GlcNAc residue). Only four direct hydrogen bonds are made between these two monosaccharide residues (Fig 5F), though a notably extensive network of water-mediated hydrogen bonds is present (Fig 5G). The recognition of this disaccharide motif is completed by the indole ring Trp386, which sits over the glycosidic bond and oriented parallel to the co-planar arrangement of the Man and GlcNAc pyranose rings such that the aromatic ring of Trp386 packs against the α-face of GlcNAc and the β-face of Man (Fig 5F).

The additional α-(1,3)- and α-(1,6)-linked mannose residues of Man_5_GlcNAc provide a number of direct hydrogen bond interactions with the protein (Fig 5F) and an extensive network of water mediated hydrogen bonds (Fig 5G). The classic CH-π interaction between aromatic sidechains and carbohydrate rings [40], such as that seen in binding the Man_1_GlcNAc core, is notably absent between the protein and the mannose residues in the glycan arms, though Trp81 does participate in hydrogen bonding with the terminal α-1,6-linked mannose.

### NgtS and EndoD contribute to growth of *S*. *pneumoniae* on a glycoconjugate

**Fig 6:**
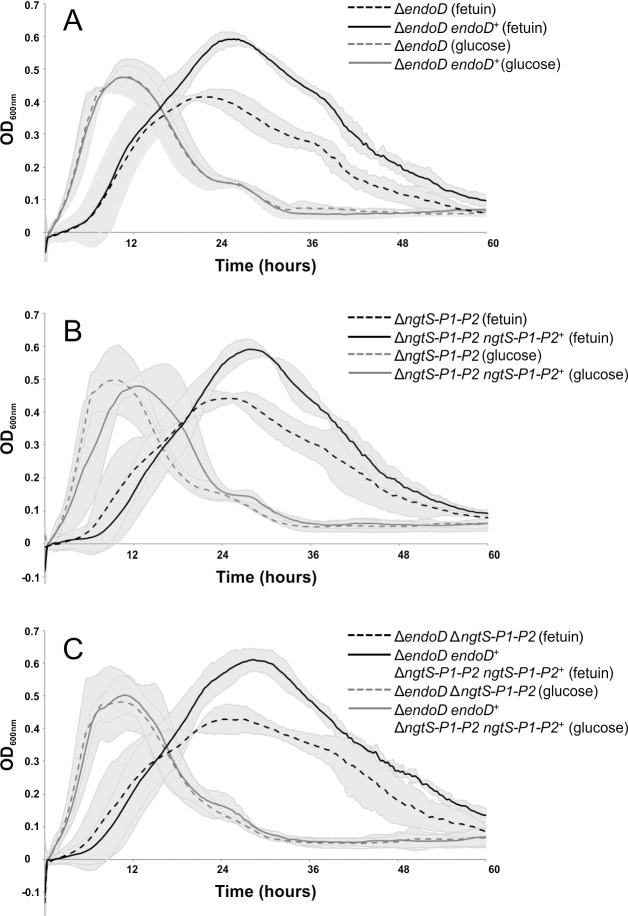
EndoD and NgtS contribute to growth of *S*. *pneumoniae* on a model glycoconjugate. Growth of deletion mutants of *S*. *pneumoniae* TIGR4 Sm^r^ on the model glycoconjugate fetuin. Deletion mutants of (A) *endoD*, (B) *ngtS-P1-P2* and (C) *endoD* plus *ngtS-P1-P2* were grown in chemically-defined medium supplemented with 20 mg ml^-1^ fetuin as the sole carbon source and compared against their genetically reconstituted strains. All OD_600nm_ readings shown are the mean from three independent experiments each performed in triplicate. Gray shading indicates the 95% confidence intervals for each strain and statistically significant differences in growth.

The growth of *S*. *pneumoniae* can be supported by the monosaccharides released during sequential deglycosylation of complex N-linked glycans by the exo-glycosidases NanA, BgaA and StrH [9,18]. This process uncaps the Man_3_GlcNAc_2_ core, which could ultimately be targeted by EndoD, therefore this enzyme may also contribute to bacterial growth on N-linked glycans. Furthermore, NgtS would also be expected to play a role in supporting growth on N-glycans through its ability to transport the glycans released by EndoD. To address this hypothesis, deletion mutants were constructed of *endoD*, the complete N-glycan ABC transporter containing NgtS and the two predicted permeases (*ngtS-P1-P2*), and both loci together. The growth of these mutant strains was tested on the model glycoprotein fetuin, which bears complex N-glycans, and glucose as a control (Fig 6). All three mutant strains were able to grow on fetuin, and at rates similar to those exhibited by the parental and genetically reconstituted strains; however, they were unable to reach the same cell density. There was no significant difference in growth on fetuin between the parental strain and any of the genetically reconstituted strains (S5 Fig), and no additive effect was observed when both loci were deleted. None of the mutants showed reduced growth on media containing glucose as the sole carbon source. The partial growth of the mutants on fetuin was expected as all three strains retain the ability to release and utilize the sialic acid, galactose and GlcNAc present in N-linked glycans through the actions of NanA, BgaA and StrH. In fact, these carbohydrates have to be sequentially removed to allow EndoD access to the Man_3_GlcNAc_2_ core. Furthermore, fetuin is also decorated with O-linked glycans that can be sequentially deglycosylated by *S*. *pneumoniae* [41]. Maximal growth on fetuin was observed between 24 and 30 hours; while this is much longer than that observed on glucose, this is not unusual for growth on less preferred carbohydrates, where maximal growth has been observed at up to 36 hours ([42–44]). Based on the data shown in Fig 6, we speculate that all the strains can initially grow on the sequentially released monosaccharides trimmed from both the complex N-glycans by NanA, BgaA and StrH and from O-glycans, therefore exhibiting the same initial growth rate. However, the deletion mutants are unable to utilize the mannose and GlcNAc present in the uncovered Man_3_GlcNAc_2_ core and so cannot reach the same total cell density. This phenotype of reduced final cell density but similar initial growth rate has been reported previously for growth of *nanA*, *bgaA*, and *strH* deletion mutants on alpha-1-acid glycoprotein ([9]). To confirm that the substrate of ABC_NG_ is not a monosaccharide released by NanA, BgaA or StrH, or a monosaccharide constituent of Man_3_GlcNAc, we tested the growth of the Δ*ngtS-P1-P2* mutant on chemically-defined medium supplemented with either 12 mM *N-*acetylneuraminic acid, galactose, GlcNAc or mannose. We saw no significant difference in growth of the Δ*ngtS-P1-P2* mutant when compared to the parental and genetically reconstituted strains (S6 Fig). Given the known specificity of EndoD, and the ability of the NgtS component of ABC_NG_ to bind N-glycans, the observation that all three mutants show very similar defects in growth on fetuin supports the hypothesis that EndoD acts to release Man_3_GlcNAc, which is then transported by ABC_NG_.

### EndoD and SpGH92, but not NgtS, contribute to virulence in a mouse model

**Fig 7:**
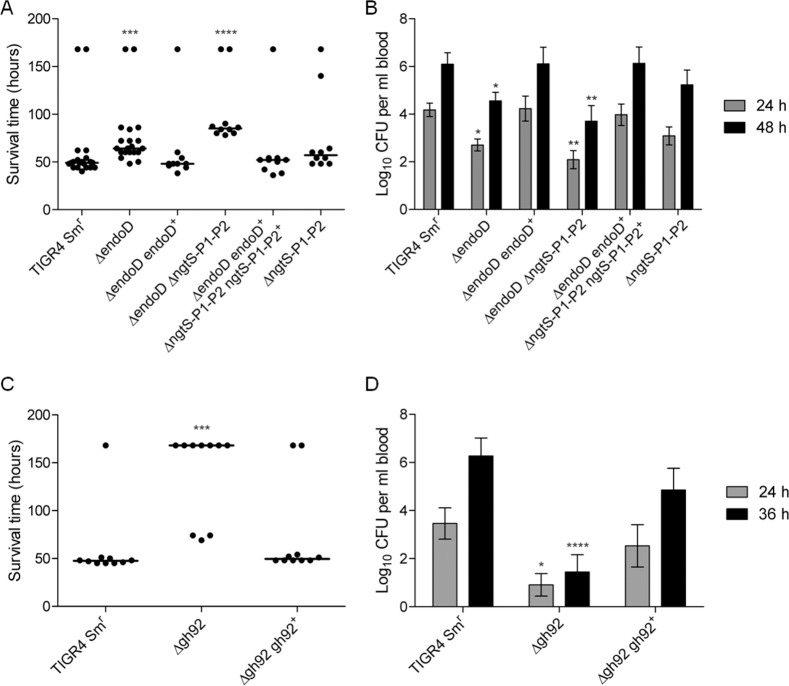
EndoD and SpGH92 contribute to virulence in a mouse model of pneumonia and sepsis. Cohorts of mice were infected intranasally with either parental TIGR4 Sm^r^, Δ*endoD*, Δ*ngtS-P1-P2*, Δ*endoD* Δ*ngtS-P1-P2*, Δ*gh92* or a genetically reconstituted strain, and monitored for survival time and bacterial counts in blood. (A) Median survival times of mice infected with parental TIGR4 Sm^r^, deletion mutants of *endoD* and/or *ngtS-P1-P2*, and genetically reconstituted strains. Symbols indicate the time that individual mice became severely lethargic and were euthanized; horizontal bars indicate the median survival time (experiment length, and therefore maximum survival time, was 168 hours). (B) Mean bacterial counts in blood 24 and 48 h post-infection with parental TIGR4 Sm^r^, deletion mutants of *endoD* and/or *ngtS-P1-P2*, and genetically reconstituted strains. Error bars represent the SEM. (C) Median survival times of mice infected with parental TIGR4 Sm^r^, Δ*gh92* and its genetically reconstituted strain. Symbols and bars are the same as in (A). (D) Mean bacterial counts in blood 24 and 36 h post-infection with parental TIGR4 Sm^r^, Δ*gh92* and its genetically reconstituted strain. Error bars represent the SEM. In all panels, asterisks indicate the level of statistical significance between medians/means when compared with the parental TIGR4 Sm^r^-infected cohort (* indicates p<0.05, ** indicates p<0.01, *** indicates p<0.001 and **** indicates p<0.0001).

In light of the contribution of EndoD and ABC_NG_ to the growth of *S*. *pneumoniae* on complex N-glycan, and the previous identification of these two proteins/protein complexes as virulence factors in signature-tagged mutagenesis studies, we assessed the contribution of EndoD and ABC_NG_ to virulence in a mouse model of pneumonia and sepsis (Fig 7). Mice were infected intranasally with parental TIGR4 Sm^r^, the single and double deletion mutants of *endoD* and *ngtS-P1-P2*, and genetically reconstituted strains, and were monitored for survival time (up to a maximum of 168 hours) and development of bacteremia. The median survival time of mice infected with Δ*endoD* (64 h ±33.2, n = 20) was significantly longer than those of the parent (49 h ±37, n = 20) and Δ*endoD endoD*^*+*^ (48 h ±38.3, n = 10) infected groups (p<0.001; Fig 7A). The deletion of both *endoD* and the *ngtS-P1-P2* loci also significantly reduced virulence as the cohort infected with the double Δ*endoD* Δ*ngtS-P1-P2* mutant (85 h ±35.8, n = 10) survived longer than the parent-infected cohort (p<0.0001). However, while the Δ*endoD* Δ*ngtS-P1-P2*-infected group survived slightly longer than those of the Δ*endoD* group, the difference was not statistically significant (p>0.05). Similarly, the Δ*ngtS-P1-P2*-infected cohort (57 h ±42.8, n = 10) did not survive significantly longer than the parent-infected group (p>0.05). The development of bacteremia showed a similar trend (Fig 7B). At 24 and 48 h post-infection, mice infected either with Δ*endoD* (log_10_ 2.7 ±0.25 and log_10_ 4.55 ±0.36 CFU ml^-1^, n = 20, for 24 and 48 h, respectively), or Δ*endoD* Δ*ngtS-P1-P2* (log_10_ 2.1 ±0.38 and log_10_ 3.7 ±0.65 CFU ml^-1^, n = 10, for 24 and 48 h, respectively) had significantly lower mean bacterial counts in their blood than the parent-infected cohort (log_10_ 4.2 ±0.28 and log_10_ 6.1 ±0.48 CFU ml^-1^, n = 20, for 24 and 48 h, respectively; p<0.05 for Δ*endoD* and p<0.01 for Δ*endoD* Δ*ngtS-P1-P2* for both time points). There was no significant difference in bacterial counts of mice infected with Δ*ngtS-P1-P2* and parental TIGR4 Sm^r^ at either 24 or 48 h post-infection (p>0.05).

The contribution of SpGH92 to virulence was also assessed by testing a Δ*gh92* mutant of *S*. *pneumoniae* TIGR4 Sm^r^ for its ability to cause disease in this same model. The median survival time of mice infected with Δ*gh92* (168 h ±46.2, n = 10) was significantly longer than those of the parent (47.5 h ±38.3, n = 10) and Δ*gh92*Δ*gh92*^*+*^ (52 h ±49.5, n = 10) infected groups (p<0.001) (Fig 7C). This increase in survival time among the mutant-infected cohort occurred in spite of the higher inoculum given to this cohort (3,520,000 CFU/mouse) compared with the parent-infected cohort (1,100,000 CFU/mouse) and, in fact, 70% of the mutant-infected cohort survived until the end of the experiment (168 hours). Consistent with the results of the survival assay, bacteremia was more severe in the cohorts infected with parental TIGR4 Sm^r^ and the genetically reconstituted strains than the Δ*gh92* infected cohort (Fig 7D). At 24 and 36 h post-infection, mice infected with Δ*gh92* (log_10_ 0.9 ±0.46 and log_10_ 1.44 ±0.72 CFU ml^-1^, n = 10, for 24 and 36 h, respectively) had significantly lower mean bacterial counts in blood than the parental (log_10_ 3.46 ±0.64 and log_10_ 6.26 ±0.74 CFU ml^-1^, n = 10, for 24 and 36 h, respectively) and Δ*gh92 gh92*^*+*^ (log_10_ 2.52 ±0.87 and log_10_ 4.85 ±0.90 CFU ml^-1^, n = 10, for 24 and 36 h, respectively) infected cohorts (p<0.05 and p<0.0001 for 24 and 36 h post-infection). There was no significant difference in bacterial counts of mice infected with Δ*gh92 gh92*^*+*^ or parental TIGR4 Sm^r^ at either 24 or 36 h post-infection (p>0.05).

## Discussion

### Building a model of N-glycan metabolism in *Streptococcus pneumoniae*

By virtue of its ability to cleave the β-(1,4)-linkage in the chitobiose core of N-linked glycans, thus releasing a glycan fragment, EndoD is expected to have a central role in the N-glycan degradation pathway of *S*. *pneumoniae*. The specificity of EndoD, however, limits its activity to N-glycans that range from Man_3_GlcNAc_2_ to Man_5_GlcNAc_2_ [20], and therefore complex N-glycans containing Gal, GlcNAc, and/or sialic acid are not substrates for EndoD. Pre-processing of complex N-glycans with the exo-glycosidases NanA, BgaA, and StrH to remove the terminal sialic acid, Gal, and GlcNAc, respectively, reduces complex N-glycans to the Man_3_GlcNAc_2_ core structure, therefore rendering them potential substrates for EndoD. This enzyme, however, is not active on mannose containing glycans (high-mannose N-glycans) larger than Man_5_GlcNAc_2_, such as those containing even a single additional α-(1,2)-mannose in Man_6_GlcNAc_2_, indicating that a large population of high-mannose glycans that may be encountered in the human body are not substrates for EndoD. We initially addressed the question of how *S*. *pneumoniae* may deal with high-mannose glycans through bioinformatics. This led to the identification of the CPL, the observed presence of the α-1,6-mannosidase SpGH125 in this locus, and the proposed functional association of EndoD with this locus, all of which led us to hypothesize that *S*. *pneumoniae* may have a more extensive capability to degrade N-linked glycans than previously thought. Of particular interest to us was SpGH92, the amino acid sequence of which places it in GH family 92, a family that contains enzymes having demonstrated activity on α(1,2), α(1,3), α(1,4), and α(1,6) mannosides. We initially targeted our studies towards SpGH92, reasoning that there was a high probability it would have activity on the mannose portions of N-linked glycans and possibly on α-1,2-linked mannose. Our results showed that *in vitro* SpGH92 is not only able to cleave α-1,2-mannobiose but that it is also able to trim these linkages in free Man_9_GlcNAc_2_ (to generate Man_5_GlcNAc_2_) or in a model glycoconjugate (RNase B). Moreover, this trimming of the glycoconjugate rendered the protein-attached N-glycan a substrate for EndoD.

These findings identify SpGH92 as one of the tools that *S*. *pneumoniae* may deploy to degrade the high-mannose arms of N-glycans, while the ability of this enzyme to generate a substrate for EndoD is consistent with the functional association of these two enzymes. As EndoD possesses a secretion signal peptide and a cell-wall anchoring LPXTG motif, it is considered part of the extracellular landscape of the bacterium. The physical location of EndoD and proposed functional association therefore presumes that SpGH92 must also be an extracellular enzyme (despite not possessing any known secretion signals) so that it can function upstream of EndoD in the N-glycan degradation pathway. We attempted to test this hypothesis by cellular fractionation followed by western blotting with an anti-SpGH92 polyclonal antibody and by activity assays using α-(1,2)-mannobiose as substrate to track the location of SpGH92 activity, but SpGH92 was not produced under our laboratory conditions in sufficient abundance to be detected in any fraction by either of these methods (S7 Fig). Nevertheless, the capacity of *S*. *pneumoniae* to non-classically secrete some proteins by an unknown mechanism is well documented [45–50] and we suggest that SpGH92 is one of these proteins. Indeed, given the specificity of this enzyme, it is the only logical physical location as *S*. *pneumoniae* has no known mechanism to endolytically release α-D-Man-(1→2)- α-D-Man containing oligosaccharides from any glycans and, therefore, if SpGH92 were an intracellular enzyme, it would apparently never encounter a substrate.

The developing model of N-glycan processing thus has substrates for EndoD generated by exo-glycosidase processing of both complex N-glycans and high-mannose N-glycans. The activity of EndoD then releases Man_3_GlcNAc to Man_5_GlcNAc glycans. These glycans must either be further depolymerized in the extracellular environment or transported *en bloc* for intracellular depolymerization. In either case, we hypothesized that *S*. *pneumoniae* must have a mechanism to import N-glycans, or fragments thereof, and given the co-occurrence of the putative ABC transporter-encoding locus SP_0090 to SP_0092 (ABC_NG_) with EndoD and the CPL, this appeared a likely candidate. As the SBPs are generally considered the specificity determinant of ABC transporters, we focused on the putative SBP, SP_0092 or NgtS, in this ABC transporter. NgtS had an affinity for Man_5_GlcNAc consistent with that observed for other SBP and carbohydrate ligand pairs [35–38]. The structure of NgtS in complex with this ligand revealed a specific network of interactions, with the suite of interactions between the reducing end GlcNAc residue and the burial of this sugar at the base of the binding site imparting specificity for the reducing end motif glycans. Indeed, our ligand binding studies indicated that the free energy (ΔG) of binding Man_1_GlcNAc, which from a structural perspective bound nearly identically to the protein as the Man_1_GlcNAc portion of Man_5_GlcNAc, was roughly 70% of that for Man_5_GlcNAc, thus providing the bulk of the binding energy and suggesting that this reducing end disaccharide motif is a key feature in ligand recognition by NgtS. The additional four α-(1,3)- and α-(1,6)-linked mannose residues of Man_5_GlcNAc contribute only ~30% of the ΔG, though this translates to a two-order of magnitude increase in the equilibrium binding constant.

Due to the lack of availability of the Man_3_GlcNAc glycan, we were unable to directly examine the binding of NgtS to this glycan. However, this glycan is an intermediate structure between Man_1_GlcNAc and Man_5_GlcNAc, both of which are NgtS ligands, and thus is also expected to be a ligand with significant affinity. Indeed, the reduced growth phenotype of the Δ*ngtS-P1-P2* mutant on fetuin provides indirect support for this. Fetuin contains only complex N-glycans that would require pre-processing by NanA, BgaA, and StrH to generate a protein-linked Man_3_GlcNAc_2_ glycan. EndoD is able to cleave peptide-linked Man_3_GlcNAc_2_ to release Man_3_GlcNAc [24] and we observed the Δ*endoD* mutant to have a phenotype of reduced growth on fetuin, providing support for this step occurring during growth on fetuin. Furthermore, the phenotype of reduced growth for the Δ*ngtS-P1-P2* mutant implies a deficiency in the ability of this mutant to transport an N-glycan substrate, which is most likely limited to the Man_3_GlcNAc released by EndoD, thus providing indirect support for the ability of NgtS to bind Man_3_GlcNAc.

Upon binding Man_5_GlcNAc, the clamshell like closing of the two NgtS domains completely engulfs the glycan. Though solvent channels connect two of the non-reducing terminal mannose residues to bulk solvent, it is unclear if the channels would be sufficient to accommodate α-(1,2)-linked additions to these arms. However, the terminal α-(1,3)-linked mannose present on the α-(1,6)-arm is completely buried thus precluding accommodation of an α-(1,2)-linked addition to this part of the glycan. However, while NgtS may be able to accommodate some forms of Man_6_GlcNAc, free N-linked glycans of any structure would be extremely rare in the host and, therefore, the ligand(s) of NgtS would most likely only be those soluble N-glycan fragments released by EndoD, which cannot be larger than Man_5_GlcNAc. Thus, the *in vivo* function of NgtS is unlikely to involve recognition of α-(1,2)-mannose modified N-glycans.

**Fig 8:**
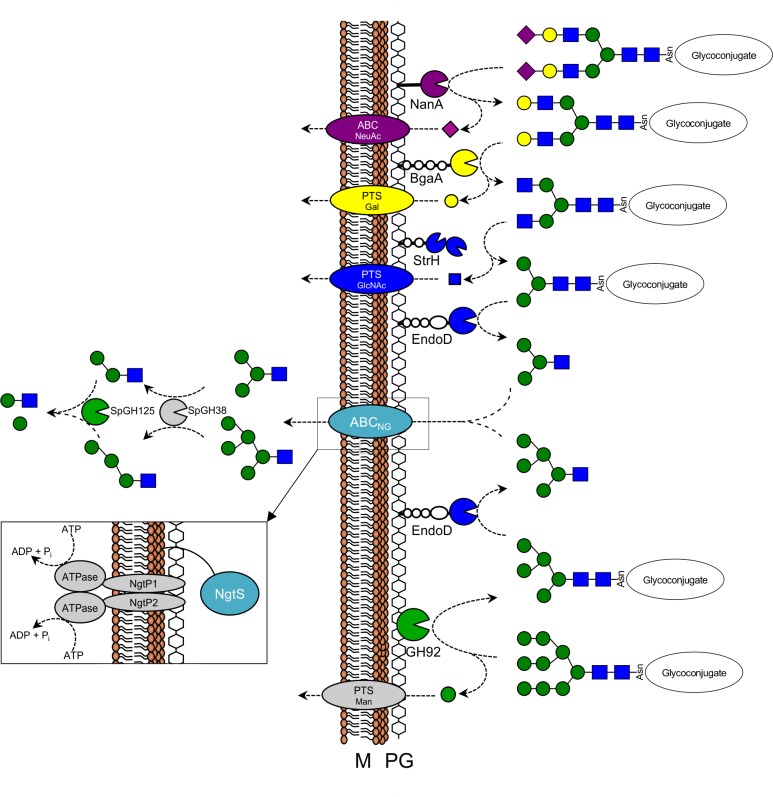
Proposed model of N-glycan metabolism in *Streptococcus pneumoniae*. The two arms of N-glycan processing—complex N-glycan (top) and high-mannose N-glycan (bottom)—are shown as graphical representations and converge at ABC_NG_. Glycosidases are colour-coded according to their known or predicted activities: sialidase (purple), β-galactosidase (yellow), β-hexosaminidase (blue) and α-mannosidase (green). StrH is shown as a multimodular complex as it contains two catalytic domains [53]. NanA, BgaA, StrH and EndoD are extracellular and all bear LPXTG cell wall anchoring motifs. Complex N-glycan is sequentially depolymerized by NanA, BgaA and StrH [18], resulting in Man_3_GlcNAc_2_, which is then released from the glycoconjugate by EndoD [20]. High-mannose N-glycan is acted on by SpGH92, to produce Man_5_GlcNAc_2_, and is then released from the glycoconjugate by EndoD. Both Man_3_GlcNAc and Man_5_GlcNAc are transported by ABC_NG_ into the cytoplasm, where further depolymerization is carried out by SpGH125 and SpGH38. Dedicated ABC and PTS transporters import the monosaccharides released by NanA, BgaA, StrH and SpGH92 [51]. The proposed architecture of ABC_NG_ is shown inset. Abbreviations: cytoplasmic membrane (M); peptidoglycan (PG).

On the basis of the results presented here and placed in the context of current knowledge, we propose an expanded model of N-glycan degradation by *S*. *pneumoniae* (Fig 8). Previously known features of this model include the sequential degradation of the complex N-glycan arms by NanA, BgaA and StrH, with the released monosaccharides likely transported into the bacterium by recently identified ABC and phosphotransferase system (PTS) transporters [51]. Direct biochemical evidence of EndoD releasing the N-glycan core after pre-processing by these exo-glycosidases has not yet been obtained; however, our observed growth phenotype of the Δ*endoD* mutant provides indirect support for this step in the pathway. The expanded model incorporates the complementary arm of this pathway that includes the processing of high mannose glycans by SpGH92 followed by release of the trimmed glycan by EndoD. *S*. *pneumoniae* possesses a mannose-specific PTS and the bacterium is able to grow when provided with free mannose [52]. However, we were unable to obtain significant growth of this bacterium on substrates for SpGH92 that would result in the release of free mannose [α-(1,2)-mannobiose and RNase B]. We were also unable to detect SpGH92 protein by western blotting or activity assay under a variety of culture conditions, including growth on these substrates, which suggests that the lack of growth on substrates of SpGH92 is likely related to low production of the enzyme *in vitro*.

The identification of an ABC transporter that possesses an SBP, NgtS, that is specific to the recognition of N-glycan fragments suggests that this is likely the convergence point of the complex and high mannose N-glycan processing sides of the N-glycan degradation pathway, with this transporter functioning to import soluble N-glycan fragments into the bacterium. ABC transporters require an associated ATPase to power the transport of solutes across the membrane. No gene encoding an ATPase was found in proximity to the genes encoding ABC_NG_ thus leading us to suggest that the ATPase utilized by this transporter may be MsmK, which has been found to function in several other *S*. *pneumoniae* carbohydrate-specific ABC transporters [42].

The intracellular component of the pathway would necessarily comprise exo-α-mannosidases to continue depolymerization of the glycan. SpGH125, which is predicted to be intracellular, has been characterized to be an exo-α-1,6-mannosidase that must act after processing by an exo-α-1,3-mannosidase [21]. By virtue of its high amino acid sequence identity (47%) to the N-glycan degrading exo-α-1,3-mannosidase SpyGH38 from *S*. *pyogenes* [32], the exo-α-1,3-mannosidase in *S*. *pneumoniae* is predicted to be SP_2143, which is a putative family 38 glycoside hydrolase (SpGH38) whose gene is also present in the CPL. Together, these enzymes are hypothesized to reduce the imported N-glycan fragment to mannose monosaccharides and Man_1_GlcNAc. The fate of this latter disaccharide is unknown as no candidate β-1,4-mannosidases have been identified in *S*. *pneumoniae* to date.

The proposed N-glycan degradation pathway thus has the elements to enable the concerted processing of glycans present on host glycoconjugates. The model, however, does not presently account for other components of the CPL, notably SpGH29 and SpGH20C. Given the predicted and known activities of these enzymes, respectively (Table 1), they may be involved in degrading the carbohydrate motifs that occasionally cap the arms of complex N-glycans, such as the histo-blood group antigens. Indeed, our recent work on SpGH20C indicates that this is a generalist exo-β-N-acetylhexosaminidase that may have the ability to process terminal β-linked GlcNAc or GalNAc residues in such capping motifs [28]. Alternatively, the function of CPL as a whole may not be specifically targeted at N-glycans and these additional GHs may target the linkages in O-glycans.

The evidence to date indicates that the N-glycan degradation pathway in *S*. *pneumoniae* plays a strong role in the host-pathogen interaction and therefore in the ability of the bacterium to cause disease. Notably, all of the enzymatic components of the proposed pathway have been identified as possible virulence factors by high-throughput screens [22,26,27,29]. Supporting this, the pneumococcal sialidase, NanA, is confirmed by several independent studies to participate in a number of aspects of the host-pathogen interaction [19,41,54,55]. More recently, BgaA was shown to function as an adhesin [56], while this enzyme and StrH both likely contribute to evasion of the innate immune response [19]. While all of these enzymes are large, multimodular proteins, whose full functions likely rely on the presence of ancillary domains, particular roles of these enzymes in the host-pathogen interaction can be modulated through the use of specific inhibitors of their catalytic activities indicating that their catalytic functions are critical. Here we have clearly demonstrated that deletion of the genes encoding EndoD or SpGH92 results in a moderate to large decrease in the ability of the microbe to cause disease in an animal model. Indeed, the effect of the Δ*gh92* mutation was particularly striking in its effect to reduce the virulence of *S*. *pneumoniae*. This observation makes SpGH92 attractive for the potential development of a therapeutic approach that targets its activity.

At present, exactly how EndoD and SpGH92 contribute to virulence is unclear. However, the observation that the *ngtS-P1-P2* deletion mutant did not show a significant virulence phenotype indicates that the metabolic assimilation of N-glycan fragments is not critical. This in turn implies that the phenotypes rendered by the Δ*endoD* and Δ*gh92* mutations result from the effects of altering the structure of glycans in the host, rather than energy liberation, and that the glycoconjugate target(s) of the EndoD and SpGH92 enzymes have a particularly important role in the host-pathogen interaction. For example, complement component C3, which is decorated with high-mannose N-glycans [57], is an important factor in the innate immune response against *S*. *pneumoniae* [58]; therefore, it may be that destruction of the glycans on this protein renders C3 unable to perform its normal role, thus providing protection of the bacterium from the immune response. Nevertheless, the relevant *in vivo* glycoconjugate targets of EndoD and SpGH92, indeed all of the enzymatic components of the N-glycan degradation pathway that influence the virulence of *S*. *pneumoniae*, remain to be conclusively identified. What appears to be conclusive given the phenotype of the Δ*gh92* mutant, which is the most profoundly attenuated GH mutant of *S*. *pneumoniae* reported to date, is that the destruction of terminal α-1,2-mannose linkages is extremely important to the interaction of this pathogen with its host in an animal model.

The genome of *S*. *pneumoniae* contains genes that encode a suite of proteins that are biochemically capable of performing the various concerted steps needed to completely depolymerize and transport all classes of N-glycans found on host glycoconjugates. Remarkably, all of the enzyme activities in the pathway are at least to some degree associated with the virulence of the bacterium, indicating a key role of these enzyme and N-glycans in the host-bacterium interaction. Moreover, the conservation of many of the genes encoding pathway components in several other streptococci, all of which are also host-adapted bacteria, suggests that this pathway may be present and possibly important in these microbes as well.

## Materials and Methods

### Ethics statement

Mouse experiments were performed under appropriate project (permit no. 60/4327) and personal (permit no. 80/10279) licenses according to the United Kingdom Home Office guidelines, and adhered to the Animals (Scientific Procedures) Act 1986. Local ethical approval was granted by the University of Leicester Animal Welfare and Ethical Review Body.

### Materials

Man_1_GlcNAc was obtained from Toronto Research Chemicals (Toronto, ON, CA) and Man_5_GlcNAc was obtained from IsoSep (Tullinge, Sweden). Man_5_GlcNAc_2_ and Man_9_GlcNAc_2_ were purchased from Carbosynth (Compton, UK). α-(1,2)-mannobiose, α-(1,3)-mannobiose, α-(1,6)-mannobiose and α-(1,3)(1,6)-mannotriose were purchased from V-Labs (Covington, LA, USA). All growth media and media components, unless otherwise stated, were from Becton, Dickinson, and Co. (Sparks, MD, USA). Unless otherwise stated, all other chemicals were from Sigma-Aldrich Co. (St. Louis, MO, USA).

### Bacterial strains and culture media

Parental and genetically modified strains of *S*. *pneumoniae* utilized in this study are described in S1 Table. Broth cultures were routinely grown at 37°C in Todd-Hewitt broth supplemented with 0.2% w/v yeast extract (THY). C media with 5% yeast extract (C+Y) pH 8.0 was used for transformations [59]. *S*. *pneumoniae* was also grown at 37°C and 5% CO_2_ overnight on tryptic soy (TS) plates with 1.5% agar that were spread with 5000 units of catalase (Worthington Biochemical Corporation, Lakewood, NJ, USA) and TS plates supplemented with 5% sheep blood. Transformants were selected on TS plates containing streptomycin (200 μg mL^-1^) or kanamycin (500 μg mL^-1^).

### Cloning

NgtS is predicted to be a cell-membrane-attached lipoprotein therefore the cloned *ngtS* gene was designed to lack the secretion signal peptide and lipobox in order to produce a soluble recombinant protein. The gene fragment encoding amino acids 25–491 of NgtS (SP_0092) was PCR-amplified from *S*. *pneumoniae* TIGR4 genomic DNA (accession no. AE005672) using the oligonucleotide primers NgtS-Fw and NgtS-Rv (S2 Table). The PCR primers used for amplification of SpGH92 (amino acids 1–694 of SP_2145) from TIGR4 were GH92-Fw and GH92-Rv. The *ngtS* insert was ligated into pET28a (Novagen, EMD Millipore Co., Darmstadt, Germany) between the 5’ *Nco*I and 3’ *Xho*I restriction sites, and the resulting plasmid (pNgtS) encoded the desired polypeptide fused to a C-terminal six-histidine tag. The *gh92* insert was ligated into pET28a between the 5' *Nhe*I and 3' *Xho*I sites, and the resulting plasmid (pGH92) encoded the desired polypeptide fused to a N-terminal six-histidine tag. The gene fragment encoding amino acids 38-1659 of EndoD (SP_0498) was amplified from TIGR4 using the primers EndoD-Fw and EndoD-Rv, and cloned into pET22b (Novagen) between the *Bam*HI and *Xho*I restriction sites using the In-Fusion HD cloning kit (Takara Clontech, Mountain View, CA, USA). The resulting plasmid (pEndoD) encoded full-length, mature EndoD with a C-terminal six-histidine tag and an N-terminal PelB leader sequence that directed the expressed protein to the periplasm. The DNA sequence fidelity of all constructs was verified by bidirectional sequencing.

### Protein expression and purification

pNgtS, pGH92 and pEndoD were transformed into the expression strain *Escherichia coli* BL21 Star (DE3). NgtS was expressed in 6 L yeast-tryptone broth overnight at 16°C with 0.5 mM isopropyl 1-thio-β-D-galactopyranoside (IPTG). Recombinant SpGH92 was produced *via* the autoinduction method [60] by incubating inoculated 1 L cultures at 37°C for 96 hours. EndoD was expressed at 37°C for 3 hours with 0.5 mM IPTG induction as described by Muramatsu *et al*. [20]. For all proteins, cells were harvested by centrifugation at 5000 × *g* for 10 minutes and ruptured by chemical lysis. The cleared supernatant of the cell lysate was loaded onto a Ni^2+^-NTA immobilized metal affinity chromatography column. Polypeptide was eluted with binding buffer (20 mM Tris, 500 mM NaCl, pH 8.0) containing increasing concentrations of imidazole (0–500 mM). Protein was concentrated and buffer-exchanged into 20 mM Tris, pH 8.0 in an Amicon stirred ultrafiltration unit (EMD Millipore Co.) with a 10,000 Da molecular weight cut-off membrane. NgtS was further purified by size exclusion chromatography using a Sephacryl S-200 column (GE Healthcare, Little Chalfont, UK) and concentrated again using an Amicon. Protein concentration was determined by UV absorbance at 280 nm using the calculated extinction coefficients of 60280 M^−1^ cm^−1^ for NgtS, 121295 M^−1^ cm^−1^ for SpGH92 and 221065 M^-1^ cm^-1^ for EndoD.

### Binding analysis

Quantitative UV difference binding studies of NgtS were performed as described previously [61] and the data analyzed using a bimolecular binding model that accounted for ligand depletion. Difference spectra were analyzed for peak and trough wavelengths, and values at the appropriate wavelengths extracted for further analysis. The peak-to-trough heights at the wavelength pairs 289.3/301.0 nm, 289.3/294.5 nm, and 282.8/287.4 nm were calculated by subtracting the trough values from the peak values, and the dilution-corrected data were plotted against total carbohydrate concentration. Data for the three wavelength pairs were analyzed simultaneously with MicroCal Origin (v.7.0). The data reported are the averages and standard deviations of three independent titrations.

ITC was performed as previously described [61], with a VP-ITC (MicroCal, Northampton, MA, USA). Briefly, protein samples were extensively dialyzed against buffer (20 mM Tris, pH 8.0) and then concentrated in an Amicon fitted with a 5000 Da molecular weight cut-off membrane. Ligand solutions were prepared by mass in buffer saved from the ultrafiltration step. Both protein and ligand solutions were filtered and degassed immediately prior to use. The binding analysis with Man_5_GlcNAc was performed with a protein concentration of 20 μM and ligand concentration of 0.5 mM, giving a C value of ~20. Titrations were performed in triplicate at 25°C. The data were fit with a single binding site model to determine K_a_, n, and ΔH. ΔS was calculated using ΔG = ΔH−TΔS. Errors represent the standard deviations of the triplicate determinations.

### SpGH92 and EndoD activity assays

SpGH92 was initially tested for activity against various oligomannoses using high performance anion exchange chromatography with pulsed amperometric detection (HPAEC-PAD). Assays were performed at 37°C in PBS (pH 7.5) with 2 mM CaCl_2_, and contained 0.5 mM substrate and 500 nM enzyme. Reactions were stopped by the addition of 140 μL 100 mM NaOH. After centrifugation at 5,000 rpm for 5 minutes, samples were analyzed by HPAEC-PAD using a Dionex ICS-3000 HPLC equipped with an ASI 100 automated sample injector and an ED50 electrochemical detector (Dionex, Sunnyvale, CA, USA) with a gold working electrode and an Ag/AgCl reference electrode. Products were analyzed using a PA-20 column set (analytical plus guard column) using an isocratic flow of 100 mM NaOH.

The activity of SpGH92 on Man_9_GlcNAc_2_ was detected by ultra performance liquid chromatography mass spectrometry (UPLC-MS). Man_9_GlcNAc_2_ at 0.6 μM was treated with 1 μM SpGH92 in 10 mM MOPS pH 7.0, 2 mM CaCl_2_ for 16 hours at room temperature, and the products were analyzed using a nanoACQUITY UPLC coupled to a Xevo G2-S QTof mass spectrometer (Waters Co., Milford, MA, USA). Glycans were separated using a 0.32 × 150 mm Hypercarb KAPPA column packed with 3 mm porous graphitized carbon particles (Thermo Fisher Scientific). The eluent flow rate was 8 μL min^-1^ with Buffer A (25 mM formic acid in 95:5 H_2_O:MeCN, raised to pH 5.0 with ammonium hydroxide) and Buffer B (25 mM formic acid in 5:95 H_2_O:MeCN, pH 5.0). Mass spectrometry was run in positive mode with settings optimized for resolution. Lockspray scans of 2 mg mL^-1^ sodium iodide in 50:50 ddH_2_O:2-propanol were collected every 10 seconds. Following injections of 1 μL samples, a gradient was run at 30°C as follows: 0–5 minutes—95% A, 5% B; 5–40 minutes—linear gradient to 20% B; 40–40.5 minutes—linear gradient back to 5% B; 40.5-45 minutes—equilibration with 5% B. Man_5_GlcNAc_2_ and Man_9_GlcNAc_2_ standards at 1 μM were used as reference samples.

The ability of SpGH92 and EndoD to deglycosylate RNase B was assessed by SDS-PAGE and intact protein MS. For both analyses, bovine pancreatic RNase B (1 mg ml^-1^) was treated with 1 μM GH92 and/or 1 μM EndoD in 10 mM HEPES pH 7.0, 2 mM CaCl_2_ at 37°C overnight. For SDS-PAGE analysis, 10 μg of each digested RNase B sample was analyzed on a 12% SDS-PAGE gel and stained with Coomassie Blue. For intact protein MS analysis, glycoforms of RNase B were identified using the UPLC-MS instrument detailed above, with C4 reversed-phase chromatography medium and eluents essentially as previously described [62].

### Crystallization of NgtS and SpGH92

Crystals were obtained at 18°C using sitting-drop vapour diffusion for screening and hanging drop vapour diffusion for optimization. NgtS crystals were grown in 29% (w/v) PEG 1500, 11% (w/v) 2-methyl-2,4-pentanediol (MPD) and 0.1 M Tris, pH 8.5 at a concentration of 15 mg mL^-1^. Crystals of NgtS in complex with Man_1_GlcNAc were obtained by co-crystallizing the protein (10 mg mL^-1^) pre-incubated with of 5 mM Man_1_GlcNAc in 23% (w/v) PEG 3350, 0.18 M sodium bromide and 0.1 M sodium citrate, pH 5.5. NgtS in complex with Man_5_GlcNAc was crystallized with protein (15 mg mL^-1^) pre-incubated with 1 mM Man_5_GlcNAc using 30% (w/v) PEG 400, 0.1 M cadmium chloride hydrate, 0.1 M sodium acetate trihydrate, pH 4.6. SpGH92 (12.5 mg ml^-1^) was crystallized in 2.25 M (NH_4_)_2_SO_4_, 0.1 M Bis-Tris, pH 5.5. The crystallization solution of apo-NgtS proved to be a cryoprotectant without any additional modifications to the solution. NgtS complexes and SpGH92 were cryoprotected in crystallization solution supplemented with 25% ethylene glycol and 30% glycerol, respectively. All crystals were flash cooled directly in a nitrogen stream at 113 K.

### Data collection and structure determination

The NgtS structure was determined by the single wavelength anomalous dispersion method using an iodide derivative. The derivative was obtained by soaking a single crystal in the crystallization solution containing 500 mM NaI for 30 seconds followed by 1 minute in the crystallization solution containing 1 M NaI. Diffraction data were collected on a home source comprising a Rigaku R-AXIS 4++ area detector coupled to a MM-002 X-ray generator with Osmic ‘‘blue” optics and an Oxford Cryostream 700. Diffraction data were processed using Mosflm and SCALA [63–65]. Phases were generated with auto-SHARP [66] using a substructure of 20 I atoms (occupancies ranging from 0.08 to 1.0), which provided a phasing power of 0.9 and figures of merit of 0.34773 and 0.16097 for acentric and centric reflections, respectively, over the full 2.1 Å resolution dataset. Autobuilding with ARP/wARP [67] using the phases generated from autoSHARP resulted in a nearly complete model.

Diffraction data for the NgtS complexes and SpGH92 were collected on beamline 9–2 and 7–1, respectively, of the Stanford Linear Accelerator Center [SLAC; Stanford Synchrotron Radiation Lightsource (SSRL)]. All diffraction data for NgtS complexes were processed using Mosflm and SCALA [63–65], and the native NgtS structure resulting from the NaI derivative structure was used as a search model to solve both complex structures using PHASER [68]. Diffraction data for SpGH92 were processed with XDS [69] and scaled with SCALA [64,65]. The *B*. *thetaiotaomicron* BT3990 structure (PDB ID: 2WVY) [33] was used as a molecular replacement model in PHASER [70], which unambiguously found four molecules in the asymmetric unit.

For all structures, the final models were obtained through iterative manual model building using COOT [71] and refinement of atomic coordinates with REFMAC [72]. The addition of water molecules was performed in COOT with FINDWATERS and manually checked after refinement. In all data sets, refinement procedures were monitored by flagging 5% of all observations as ‘‘free” [73]. Model validation was performed with MOLPROBITY [74].

### Molecular weight estimation of SpGH92

The oligomeric state of SpGH92 in solution was determined by size-exclusion chromatography and dynamic light scattering (DLS). A Hi-Prep 16/60 Sephacryl S-500 HR column (GE Healthcare) was calibrated using thyroglobulin (6 mg), ferritin (3.75 mg) and aldolase (3.1 mg) from the Gel Filtration HMW Calibration Kit (GE Healthcare), and β-amylase (4.1 mg) from the Gel Filtration Marker Kit (Sigma-Aldrich). Blue dextran (2.5 mg) was used to determine the void volume. The column was run at 1 ml/min in 20 mM Tris pH 8.0, 100 mM NaCl and 500 μl of each standard was injected using a 1 ml loop. Protein elution was followed at *A*_280nm_ and a standard curve generated from the elution volumes as detailed in the GE Healthcare Size Exclusion Chromatography: Principles and Methods handbook. SpGH92 (4 mg) was loaded onto the column in the same way and its molecular weight estimated from the standard curve. DLS was performed on SpGH92 following size-exclusion chromatography using a DynaPro plate reader (Wyatt Technology, Santa Barbara, CA, USA) with a wavelength of 833.78 nm at 25°C. Eight samples of 40 μL were loaded and 10 acquisitions of 5 s were performed for each sample. Data were collected and analyzed using the accompanying Dynamics V7.1 software under the globular proteins model.

### SpGH92 localization by western blotting and FACE

Attempts were made to detect SpGH92 in cellular fractions of TIGR4 Sm^r^ by western blotting and fluorophore-assisted carbohydrate electrophoresis (FACE). For western blotting, TIGR4 Sm^r^ was grown in AGCH media containing 1% mannose, glucose or galactose at 37°C for 10 hours, pelleted and resuspended in Laemmli buffer. Samples were heated, run on a 10% SDS-PAGE gel and western blotting performed as previously described [28] using a 1/4000 dilution of rabbit antiserum raised against purified recombinant SpGH92. For FACE analysis, TIGR4 Sm^r^ was grown in 50 mL AGCH medium with 1% glucose to an OD_600_ of 0.6, then pelleted and resuspended in AGCH containing no sugar and incubated for a further 30 minutes (in an attempt to induce expression of SpGH92). The cells were then pelleted again, and the supernatant retained as the extracellular fraction and concentrated 100-fold using an Amicon ultrafiltration cell fitted with a 10 kDa MWCO membrane. The pelleted cells were washed with 50 mM Tris-HCl pH 7.5, resuspended in cell wall digestion buffer [75] and incubated at 37°C with gentle shaking for 2 hours to generate protoplasts. The protoplasts were then pelleted and the supernatant retained as the cell wall fraction. The cytoplasmic fraction was obtained by gently washing the protoplasts with 50 mM Tris-HCl pH 7.5, 30% sucrose, resuspending and lysing them in 50 mM Tris-HCl pH 7.5, pelleting the protoplast membranes at 20,000 rpm for 30 minutes and retaining the supernatant. Finally, the membrane fraction was obtained by solubilizing the membranes in 50 mM Tris-HCl pH 7.5, 0.05% Triton as previously described [76]. The different fractions were kept on ice and 10 μL of each (25 μg total protein) was added to 10 μg α-(1,2)-mannobiose in PBS pH 7.5, 2 mM CaCl_2_; a reaction containing 1 μM recombinant SpGH92 was included as a control. Reactions were incubated at 37°C for 48 hours then dried and labelled with the fluorophore 8-aminonaphthalene-1,3,6-trisulfonic acid (ANTS) in the presence of sodium cyanoborohydride. Labelled glycans were separated on a 35% polyacrylamide gel and visualized under UV light.

### Construction of *S*. *pneumoniae* deletion mutants

All mutants were constructed using the Janus cassette selection system [77]. This method requires two rounds of transformation. The first introduced a Janus cassette containing genes encoding for kanamycin resistance and streptomycin sensitivity (*rpsL*^+^) into the genome of streptomycin-resistant (Sm^r^) *S*. *pneumonia*e in place of the region to be deleted. DNA fragments flanking the region to be deleted were amplified (primers 1 and 2 and primers 4 and 6; S2 Table) and sequentially joined to the Janus cassette PCR product (primers Janus-Fw and Janus-Rv) using a variation of splicing by overlap extension (SOE) PCR [9], first described by Horton *et al*. [78]. All genomic DNA was prepared as previously described [79]. A high-fidelity proofreading polymerase (Phusion; New England Biolabs, Ipswich, MA, USA) was used to minimize PCR-generated errors. The Janus construct was transformed into *S*. *pneumoniae* and the transformants were selected for on kanamycin and confirmed by PCR using primers 7 and 8, which flank the mutant construct. The second round of transformation replaced the Janus cassette with an engineered segment of DNA consisting of the fragments flanking the deleted region. The fragments were generated using primers 1 and 3 (upstream fragment) and 5 and 6 (downstream fragment) and spliced together via SOE. The unmarked mutants were confirmed with primers flanking the construct (primers 7 and 8) and bidirectional sequencing. The genetically reconstituted strains were generated by transforming the final mutants with the corresponding Janus construct and then subsequently with parental PCR products. Genetic reconstitution was confirmed by PCR and sequencing with primers flanking the construct (7 and 8). The Janus system of mutant generation is designed to generate unmarked, non-polar mutants; however, as the genes encoding ABC_NG_ (SP_0090–92) and *endoD* (SP_0498) have genes downstream which may be in the same operon, we demonstrated that the mutations had no effect on the transcription of the distal gene by reverse transcriptase RT-PCR (S8 Fig; see below for methods). cDNA was amplified with primers designed within the gene distal to the mutation: primers EndoD-9 and EndoD-10 for the *endoD* mutant (SP_0499) and primers ABC_NG_-9 and ABC_NG_-10 for the *ngtS-P1-P2* mutant (SP_0095; S2 Table). *aroE* was used as a housekeeping gene to confirm similar levels of cDNA in all preparations.

### *S*. *pneumoniae* growth assays

Chemically defined media (CDM) was prepared essentially as previously described [80], without the addition of carbohydrate. The CDM buffer was made at 2.5 × concentration to allow addition of sufficient carbohydrate to support bacterial growth. The medium was supplemented with no sugar, glucose (12 mM), fetuin (20 mg mL^-1^), GlcNAc (12 mM), galactose (12 mM), mannose (12 mM) or *N*-acetylneuraminic acid (12 mM). *S*. *pneumoniae* strains were grown in THY to an optical density at 600 nm (OD_600_) = 0.3 ± 0.005, and 2 mL aliquots were washed and resuspended in 130 μL PBS plus 130 μL catalase (50,000 U mL^-1^). Twenty microliter aliquots of bacterial suspensions or PBS/catalase (no bacteria control) were transferred to 180 μL CDM supplemented with the appropriate carbon source. Medium supplemented with glucose served as a positive control, demonstrating in each experiment that bacteria were viable and that mutant strains showed no general growth defect relative to their parent strain. Medium with no added carbohydrate served as a negative control. Growth plates were incubated at 37°C for 60 hours in a Synergy HT plate reader (BioTek, Winooski, VT, USA) and the OD_600_ were read every 20 minutes. All data were adjusted to a path-length of 1 cm. As no increase in optical density was observed for any bacterial strain on no-carbohydrate medium, at each time point the average of results from triplicate wells was subtracted from results from all other wells containing the same bacterial strain. Data from at least three independent experiments were averaged, and the 95% confidence interval was calculated for each time point.

### RNA preparation and reverse transcriptase RT-PCR

RNA was isolated by an acid-phenol extraction, with modifications for *S*. *pneumoniae*. Bacteria were grown in THY until samples reached OD_600_ = 0.3 ± 0.005. A 10 mL culture was combined with 10 mL acid phenol and 100 μL 10% SDS and incubated at 90°C until the phases merged. Samples were then cooled on ice and centrifuged at 4°C for 20 minutes at 3200 × *g*. Two additional extractions were performed, first with equal volumes of 1:1 acid phenol:choloroform and subsequently with chloroform. RNA was precipitated with 10 mL isopropanol and 1 mL 3 M sodium acetate at -20°C overnight. RNA was concentrated and washed twice with 70% ethanol and then vacuum dried. The resulting RNA was resuspended in 100 μL dH_2_O, quantified and adjusted to 100 μg μL^-1^ to allow for a final reaction volume of 50 μL for DNase treatment. Nucleic acid was incubated with 5 μL DNase I buffer, 3 μL DNase I (New England Biolabs) and 1 μL Super RNaseIN (Promega) at 37°C for 1 hr. Cleanup of RNA was performed with a Qiagen RNeasy mini kit as per manufacturer’s instructions. DNase- and RNase-free reagents were used throughout. cDNA was generated with SmartScribe reverse transcriptase according to the manufacturer’s recommendations (New England Biolabs). Parallel samples were processed without the addition of reverse transcriptase as a negative control.

### *In vivo* analysis of pneumococcal strains

*In vivo* analysis of all the pneumococcal strains used ten-week-old female MF1 outbred mice (Charles River, Margate, UK). Where appropriate, the procedures were carried out under anesthetization with isoflurane. Mice were kept in individually ventilated cages in a controlled environment and were frequently monitored after infection to minimize suffering. For infections, a standard inoculum was prepared for each strain. Briefly, for infections involving all strains except the Δ*gh92* and Δ*gh92 gh92*^*+*^ strains, pneumococci were grown overnight in Brain-Heart Infusion (BHI), pelleted by centrifugation and re-suspended in PBS (pH 7.0). A 100 μL aliquot of this suspension was administered into the peritoneal cavity. Once the animals started displaying signs of disease, blood was collected by cardiac puncture under deep anesthesia to inoculate 10 mL BHI. After overnight growth, the bacterial pellet was used to inoculate BHI containing 20% calf serum, and the growth was allowed to continue until late exponential phase (OD_500_ 1.4–1.6). At this stage, growth was stopped and the aliquots of bacteria were stored at -80°C until required. For infections involving the Δ*gh92*, Δ*gh92 gh92*^*+*^ and parental TIGR4 Sm^r^ control strains, a standard inoculum was prepared for each strain by inoculating fresh BHI serum broth (80% (v/v) BHI and 20% (v/v) BSA) with an overnight culture of pneumococci. When the OD_500_ reached 1.6, bacteria were stored at −80°C in small aliquots until needed. Immediately before infection, bacteria were diluted to 2 × 10^7^ CFU/mL in PBS (pH 7.0).

To assess the virulence of pneumococcal strains, mice were lightly anesthetized with 3% (v/v) isoflurane over oxygen and an inoculum of 50 μL containing approximately 1 × 10^6^ CFU in PBS was given drop-wise into the nostrils as described previously [81, 82]. After infection, the inoculum dose was confirmed by viable counting on blood agar plates to determine the actual administered dose. Mice were monitored for disease signs (progressively starry coat, hunched, and lethargic) for 7 days, and disease signs were evaluated independently by two experienced operators. When mice were lethargic, they were culled. Therefore, time to reach lethargic state has been defined as the “survival time.” Mice that were alive 7 days after infection were deemed to have survived the infection.

To monitor the development of bacteremia in each mouse, approximately 20 μL venous blood was obtained from mice at pre-determined time points after infection. Viable counts in blood were determined by serial dilution in sterile PBS and plating onto blood agar plates supplemented with 5% (v/v) defibrinated horse blood. Survival times were calculated using GraphPad Prism software and analyzed by the Mann-Whitney U test. Bacterial counts in blood were analyzed by an analysis of variance followed by the Bonferroni post-test. Statistical significance was considered to be a *P* value of <0.05.

## Supporting Information

S1 Fig Schematic depiction of the structure and composition of N-glycan. (A) Simplified depiction of complex N-glycan, with the common N-glycan core (Man_3_GlcNAc_2_) boxed. (B) Simplified depiction of high-mannose N-glycan (Man_9_GlcNAc_2_).
(TIF)

S2 Fig Organization and conservation of a Carbohydrate Processing Locus (CPL) and other accessory proteins predicted to be associated with N-glycan processing. Conservation of the CPL and accessory ORFs in streptococci and other members of the Firmicutes. Percentages shown above ORFs represent the amino acid sequence identity to the *S*. *pneumoniae* homolog (shown in Fig 1). Color coding and naming of ORFs is the same as in Fig 1. Additional ORFs found in the loci of *Lactococcus lactis*, *Streptococcus pasteurianus* and *Lactobacillus plantarum* encode for transcriptional regulators (Reg), sensors and a truncated transposase (Trans).
(TIF)

S3 Fig SDS-PAGE gel of glycosylated and deglycosylated RNase B following treatment with SpGH92 and EndoD. The change in size of RNase B following deglycosylation by SpGH92 and EndoD is shown. Native RNase B consists of Man_5_-Man_9_ glycoforms and has a mean size of approx. 18 kDa. Upon treatment with SpGH92 alone, these glycoforms are uniformly trimmed down to Man_5_. EndoD is only able to cleave the chitobiose core of the Man_5_ glycoforms, therefore the Man_6_-Man_9_ glycoforms remain intact and two bands for RNase B are observed (glycosylated and deglycosylated). Together, SpGH92 and EndoD fully deglycosylate RNase B.
(TIF)

S4 Fig Molecular weight estimation of SpGH92 by gel filtration. (A) Protein standards of known molecular weight were used to calibrate a HiPrep 16/60 Sephacryl S-500 HR column: thyroglobulin (669 kDa), ferritin (440 kDa), β-amylase (200 kDa) and aldolase (158 kDa). (B) Gel filtration trace of SpGH92 on the HiPrep 16/60 Sephacryl S-500 HR column. (C) Linear regression analysis of the protein standards. K_av_ values were calculated from the elution volume, bed volume and void volume (as determined by the elution volume of blue dextran) as detailed in the manufacturer’s handbook. According to its elution volume, the K_av_ of SpGH92 was 0.599 which equates to a molecular weight of 333.65 kDa.
(TIF)

S5 Fig Growth of parental and genetically reconstituted strains on fetuin. Growth profiles on fetuin of (A) the parental strain and *endoD* genetically reconstituted strain, (b) the parental strain and *ngtS-P1-P2* genetically reconstituted strain and (c) the parental strain and double mutant genetically reconstituted strain grown in chemically defined medium supplemented with 20 mg/ml fetuin as the sole carbon source. Growth was measured by optical density at 600 nm. Data for a no-carbohydrate control were subtracted from each dataset. Data points are the means from three independent experiments performed in triplicate. Gray shading indicates the 95% confidence intervals for each strain and statistically significant differences in growth.
(TIF)

S6 Fig Growth of parental strain, *ngtS-P1-P2* mutant and genetically reconstituted strain on monosaccharides. Growth of the parental strain, *ngtS-P1-P2* mutant and genetically reconstituted strain was tested on chemically defined medium supplemented with 12 mM (A) N-acetylglucosamine, (B) galactose (C) mannose or (D) sialic acid as the sole carbon source. Growth was measured by optical density at 600 nm. Data for a no-carbohydrate control were subtracted from each dataset. Data points are the means from three independent experiments performed in triplicate. Gray shading indicates the 95% confidence intervals for each strain and statistically significant differences in growth.
(TIF)

S7 Fig Attempts to detect SpGH92 in TIGR4 Sm^r^ cell lysate. (A) Western blot analysis of SpGH92 levels in TIGR4 Sm^r^ grown on different carbohydrates using rabbit antiserum raised against purified recombinant SpGH92. Lane 1–4: 100, 50, 10 and 1 ng recombinant SpGH92, respectively; lane 5: protein size ladder; lane 6–8: cell lysate from cells grown on mannose, glucose and galactose, respectively. No SpGH92 was detected in cell lysates; as a positive control, the same samples were blotted with an anti-GH20C antibody and GH20C was detected in the glucose-grown cell lysate as previously described [28]. (B) Screen of TIGR4 Sm^r^ cellular fractions for SpGH92 activity by fluorophore-assisted carbohydrate electrophoresis (FACE). TIGR4 Sm^r^ cells were fractionated into extracellular (Ex), cell wall (CW), cytoplasmic (Cyto) and membrane (Mem) fractions, incubated with α-(1,2)-mannobiose, and the resulting glycans labelled with a fluorophore; activity of recombinant SpGH92 was also included as a control. Fractions alone were also labelled with fluorophore and showed some background labelling (see last three lanes). SpGH92 activity could not be detected in any of the fractions.
(TIF)

S8 Fig Reverse transcriptase RT-PCR showing no polar effects of gene deletions. The Δ*endoD*, Δ*ngts-P1-P2* and double mutations had no effect on the transcription of the distal gene by reverse transcriptase RT-PCR. cDNA was amplified with primers designed within the gene distal to the mutation. + and–indicate the presence and absence of reverse transcriptase in the cDNA synthesis reaction. *aroE* is a housekeeping gene used to confirm similar levels of cDNA in all preparations. gDNA is genomic DNA while NT is a no template control.
(TIF)

S1 Table *S*. *pneumoniae* strains used in this study. (DOCX)

S2 Table Primers used in this study. (DOCX)
