# Supplementary figures and images for: Molecular Characterization of N-glycan Degradation and Transport in Streptococcus pneumoniae and Its Contribution to Virulence
Source: PLoS Pathog. 2017 Jan 5;13(1):e1006090. doi: 10.1371/journal.ppat.1006090 (PMC5215778; doi:10.1371/journal.ppat.1006090)

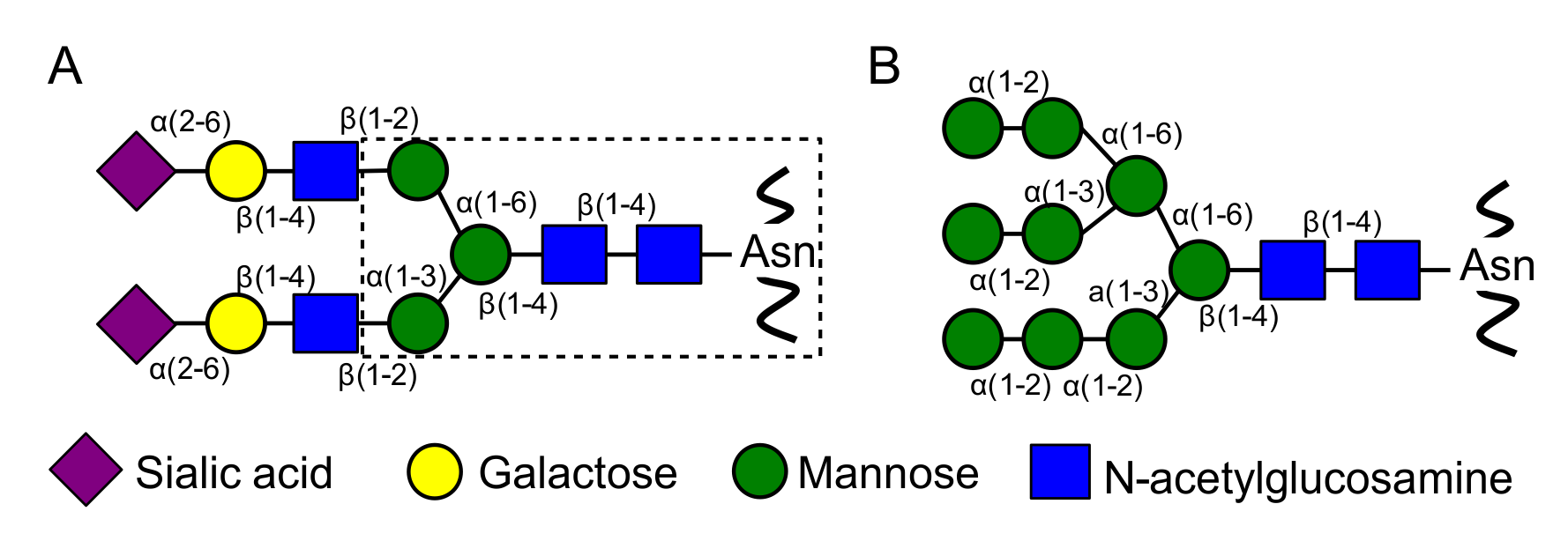

Supplement: S1 Fig — (A) Simplified depiction of complex N-glycan, with the common N-glycan core (Man3GlcNAc2) boxed. (B) Simplified depiction of high-mannose N-glycan (Man9GlcNAc2). (TIF) [file ppat.1006090.s001.tif]

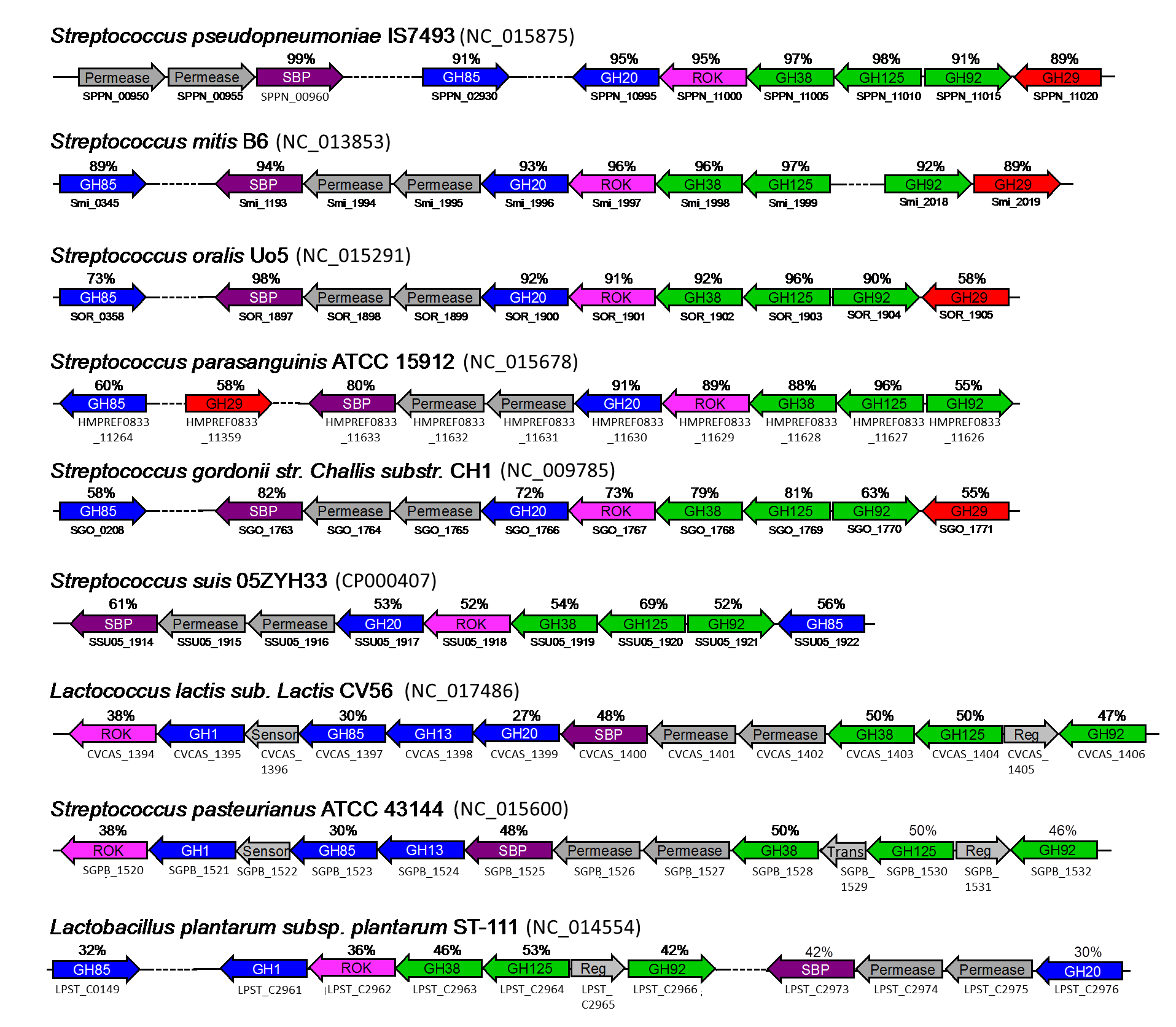

Supplement: S2 Fig — Conservation of the CPL and accessory ORFs in streptococci and other members of the Firmicutes. Percentages shown above ORFs represent the amino acid sequence identity to the S. pneumoniae homolog (shown in Fig 1). Color coding and naming of ORFs is the same as in Fig 1. Additional ORFs found in the loci of Lactococcus lactis, Streptococcus pasteurianus and Lactobacillus plantarum encode for transcriptional regulators (Reg), sensors and a truncated transposase (Trans). (TIF) [file ppat.1006090.s002.tif]

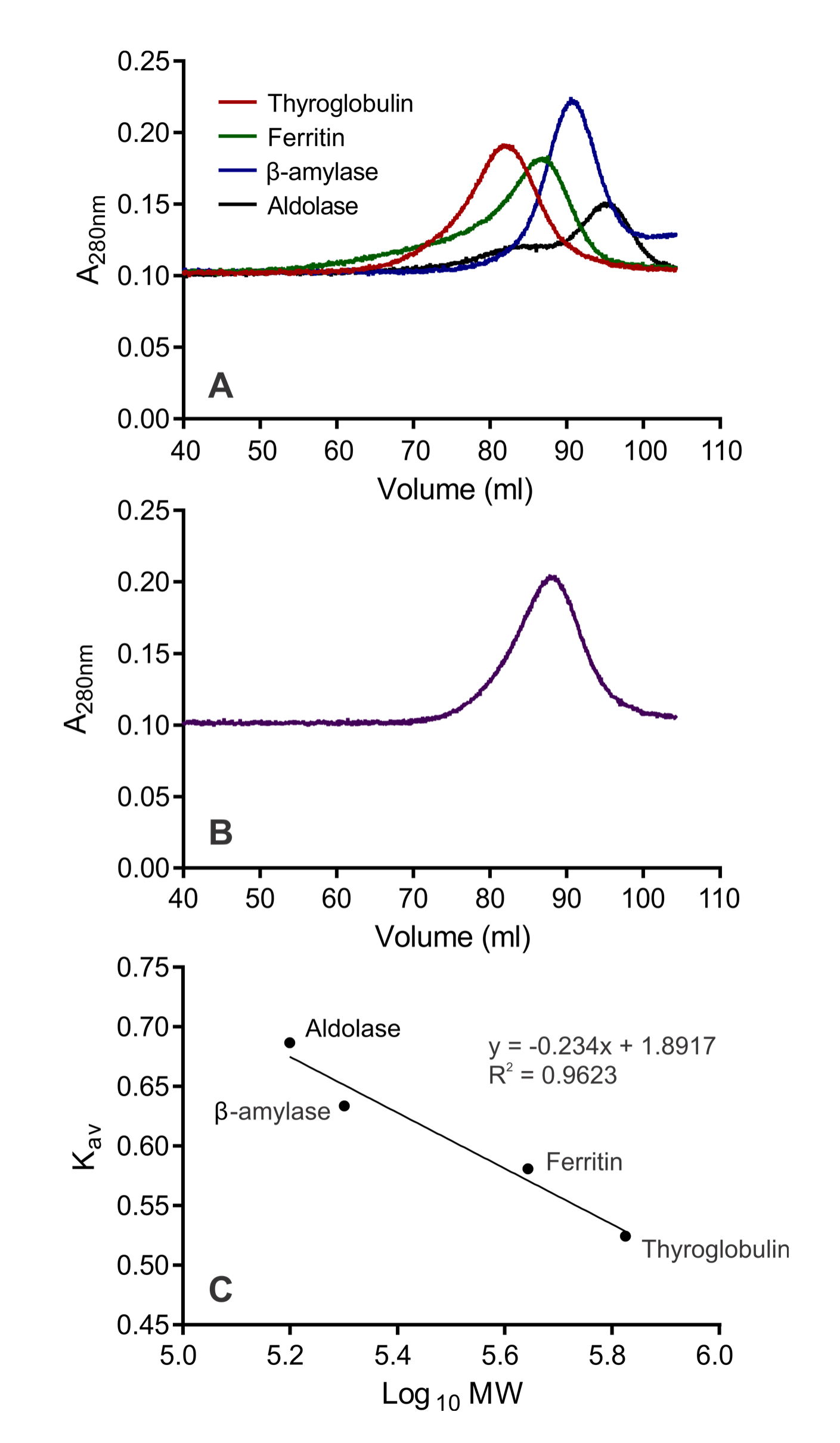

Supplement: S4 Fig — (A) Protein standards of known molecular weight were used to calibrate a HiPrep 16/60 Sephacryl S-500 HR column: thyroglobulin (669 kDa), ferritin (440 kDa), β-amylase (200 kDa) and aldolase (158 kDa). (B) Gel filtration trace of SpGH92 on the HiPrep 16/60 Sephacryl S-500 HR column. (C) Linear regression analysis of the protein standards. Kav values were calculated from the elution volume, bed volume and void volume (as determined by the elution volume of blue dextran) as detailed in the manufacturer’s handbook. According to its elution volume, the Kav of SpGH92 was 0.599 which equates to a molecular weight of 333.65 kDa. (TIF) [file ppat.1006090.s004.tif]

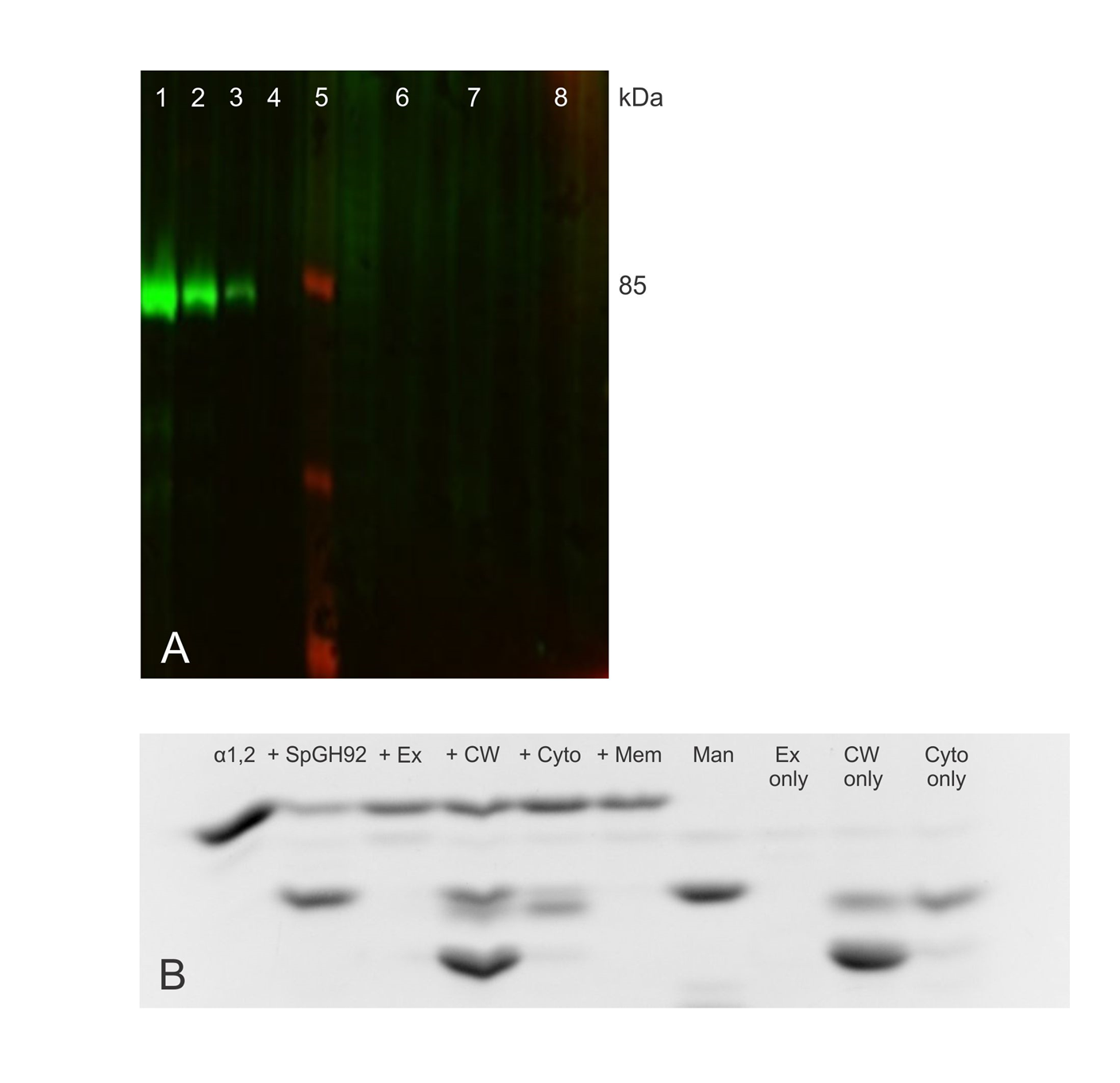

Supplement: S7 Fig — (A) Western blot analysis of SpGH92 levels in TIGR4 Smr grown on different carbohydrates using rabbit antiserum raised against purified recombinant SpGH92. Lane 1–4: 100, 50, 10 and 1 ng recombinant SpGH92, respectively; lane 5: protein size ladder; lane 6–8: cell lysate from cells grown on mannose, glucose and galactose, respectively. No SpGH92 was detected in cell lysates; as a positive control, the same samples were blotted with an anti-GH20C antibody and GH20C was detected in the glucose-grown cell lysate as previously described [28]. (B) Screen of TIGR4 Smr cellular fractions for SpGH92 activity by fluorophore-assisted carbohydrate electrophoresis (FACE). TIGR4 Smr cells were fractionated into extracellular (Ex), cell wall (CW), cytoplasmic (Cyto) and membrane (Mem) fractions, incubated with α-(1,2)-mannobiose, and the resulting glycans labelled with a fluorophore; activity of recombinant SpGH92 was also included as a control. Fractions alone were also labelled with fluorophore and showed some background labelling (see last three lanes). SpGH92 activity could not be detected in any of the fractions. (TIF) [file ppat.1006090.s007.tif]
