# Supplementary material for: Molecular Characterization of N-glycan Degradation and Transport in Streptococcus pneumoniae and Its Contribution to Virulence
Source: PLoS Pathog. 2017 Jan 5;13(1):e1006090. doi: 10.1371/journal.ppat.1006090 (PMC5215778; doi:10.1371/journal.ppat.1006090)
Supplement: S1 Table — (DOCX) [file ppat.1006090.s009.docx]

**S1 Table. *S. pneumoniae* strains used in this study**

| **Strain name** | **Characteristics/genotype** | **Source/**  **reference** |
| --- | --- | --- |
| TIGR4 Sm^r^ | TIGR4 with K56T mutation in *rpsL* conferring Sm^r^ | (1) |
| TIGR4 Δ*endoD* | TIGR4 Sm^r^ with SP_0498 deleted | This study |
| TIGR4 Δ*endoD endoD^+^* | TIGR4 Sm^r^ with SP_0498 deleted and reinserted | This study |
| TIGR4 Δ*ngtS-P1-P2* | TIGR4 Sm^r^ with SP_0090-0092 deleted | This study |
| TIGR4 Δ*ngtS-P1-P2*  *ngtS-P1-P2^+^* | TIGR4 Sm^r^ with SP_0090-0092 deleted and reinserted | This study |
| TIGR4 Δ*endoD* Δ*ngtS-P1-P2* | TIGR4 Sm^r^ with SP_0498 and SP_0090-0092 deleted | This study |
| TIGR4 Δ*endoD* Δ*ngtS-P1-P2 endoD^+^ ngtS-P1-P2^+^* | TIGR4 Sm^r^ with SP_0498 and SP_0090-0092 deleted and reinserted | This study |
| TIGR4 Δ*gh92* | TIGR4 Sm^r^ with SP_2145 deleted | This study |
| TIGR4 Δ*gh92 gh92^+^* | TIGR4 Sm^r^ with SP_2145 deleted and reinserted | This study |

Abbreviation: Streptomycin resistance (Sm^r^)

**Supporting Reference**

1. Bender MH, Weiser JN. The atypical amino-terminal LPNTG-containing domain of the pneumococcal human IgA1-specific protease is required for proper enzyme localization and function. Mol Microbiol. 2006 Jul;61(2):526–43.
