# Supplementary material for: Molecular Characterization of N-glycan Degradation and Transport in Streptococcus pneumoniae and Its Contribution to Virulence
Source: PLoS Pathog. 2017 Jan 5;13(1):e1006090. doi: 10.1371/journal.ppat.1006090 (PMC5215778; doi:10.1371/journal.ppat.1006090)
Supplement: S2 Table — (DOCX) [file ppat.1006090.s010.docx]

**S2 Table. Primers used in this study**

| **Primer use** | **Primer name** | **Primer sequence (5'-3')** |
| --- | --- | --- |
| **Cloning** | NgtS-Fw | CATATCCCATGGGAAATTTGACAGGTAACAG |
|  | NgtS-Rv | GGTGGTCTCGAGCTTTTTGTTTTTCAAGAATTCATC |
|  | GH92-Fw | CATATCGCTAGCATGAAACCACTAC |
|  | GH92-Rv | GGTGGTCTCGAGTTAGTGACTTGGTAAC |
|  | EndoD-Fw | ATCGGAATTAATTCGGAAGAAACTGCAGTTCCTGAAAATAGC |
|  | EndoD-Rv | TGGTGGTGGTGCTCGAAATCTTTATCTTCTCTTTTCCGTCTC |
| **Mutant construction** | Janus-Fw | CCGTTTGATTTTTAATGGATAATG |
|  | Janus-Rv | GGGCCCCTTTCCTTATGCTT |
|  | EndoD-1 | GTAAACCGTCTCCCATGGTCTTGATG |
|  | EndoD-2 | CATTATCCATTAAAAATCAAACGGAGCAGTTTCAGATGCTGTCGCTAC (1) |
|  | EndoD-3 | AGCAGTTTCAGATGCTGTCGCTACTC |
|  | EndoD-4 | AAGCATAAGGAAAGGGGCCCCCTAAATTGGAAGTTCAAGAG (2) |
|  | EndoD-5 | GAGTAGCGACAGCATCTGAAACTGCTCCTAAATTGGAAGTTCAAGAG (3) |
|  | EndoD-6 | TTATCAGATTCTTCTTTCACACGTCC |
|  | EndoD-7 | ACTGTTACACCAGAACTAGACTG |
|  | EndoD-8 | TCCAATTCAGCACCACGAGTGAC |
|  | ABC_NG_-1 | AGCCAGTCAGCAACTAAG |
|  | ABC_NG_-2 | CATTATCCATTAAAAATCAAACGGAAGAATAGAATCAACCAGAG (1) |
|  | ABC_NG_-3 | TCACGAACGAACCAAGTATCAAGAATAGAATCAACCAGAG (4) |
|  | ABC_NG_-4 | AAGCATAAGGAAAGGGGCCCGATACTTGGTTCGTTCGTGA (2) |
|  | ABC_NG_-5 | GATACTTGGTTCGTTCGTGA |
|  | ABC_NG_-6 | TTCGTAGGCACCTTCAGAT |
|  | ABC_NG_-7 | TGCTTCCCTAGAGTCCATT |
|  | ABC_NG_-8 | CTACACTAGTATACCTGCTT |
|  | GH92-1 | GATACCCGCTTTGGAACTACC |
|  | GH92-2 | CATTATCCATTAAAAATCAAACGGTTGCAGGGCAGTGAACATCG (1) |
|  | GH92-3 | TTGCAGGGCAGTGAACATCG |
|  | GH92-4 | AAGCATAAGGAAAGGGGCCCAAAGCCTGTCAAGATGCTCC (2) |
|  | GH92-5 | CGATGTTCACTGCCCTGCAAAAAGCCTGTCAAGATGCTCC (5) |
|  | GH92-6 | TTGGTAACCAGCTGAGGGTG |
|  | GH92-7 | TTTGAGACAGAAAATACATACAC |
|  | GH92-8 | TCTATCTGAACCTAACGGTGG |
| **RT-PCR** | EndoD-9 | CACTCGTTACGAAGATGTTGA |
|  | EndoD-10 | GATTTCGATACCTTGTGCTTTG |
|  | ABC_NG_-9 | CCGTATCCTAGTCGCTGAC |
|  | ABC_NG_-10 | CCTTGTTATCACGGACCCATT |
|  | *aroE*-up | GCCTTTGAGGCGACAGC |
|  | *aroE*-dn | TGCAGTTCA(G/A)AAACAT(A/T)TTCTAA |

Underlining indicates reverse complement sequence of primer Janus-Fw (1), Janus-Rv (2), EndoD-3 (3), ABC_NG_-5 (4) and GH92-3 (5).
